# Assessing Fluorosulfonyl Pentafluorooxosulfate (FSO_2_–OSF_5_) Reservoir Capacity: Selective SOF_4_, SO_2_F_2_, and [OSF_5_]^–^ Anion Release

**DOI:** 10.1002/anie.202510796

**Published:** 2025-07-30

**Authors:** Alexandre Millanvois, Carlota Bahri, Thomas Drews, Simon Steinhauer, Sebastian Riedel

**Affiliations:** ^1^ Fachbereich Biologie, Chemie Pharmazie Institut für Chemie und Biochemie – Anorganische Chemie Freie Universität Berlin Fabeckstr. 34/36 14195 Berlin Germany

**Keywords:** Click chemistry, Pentafluorooxosulfate salts (OSF_5_), Storage, SuFEx, Thionyl tetrafluoride

## Abstract

Herein, the investigation of inorganic reagents containing the pentafluorooxosulfate (OSF_5_) moiety is presented. First, the gram‐scale preparation of the reactive yet stable pentafluorosulfur hypofluorite (FOSF_5_) as well as its spectroscopic data are reported. Then, the preparation and full characterization of the corresponding fluorosulfonyl pentafluorooxosulfate (FSO_3_SF_5_, *FSPO*) by the fluoro‐pentafluorooxosulfanylation of SO_2_ are highlighted. This liquid reagent (b.p. 39.6 °C) was later successfully used for modulable release of [cat][OSF_5_] ion‐pair. The selective, *in‐solution* release of SOF_4_ and SOF_4_/SO_2_F_2_ species was also demonstrated. Finally, the SuFEx reactivity was investigated and showed interesting reservoir ability. Indeed, we report the one‐pot synthesis of sulfonyl fluoride (‐SO_2_F) and sulfurimidoyl fluoride (─N═S(O)F_2_) species.

## Graphical abstract

**Tidying away gases and anions**. The complementary storage & *in‐solution* release of tetrafluoride (SOF_4_), sulfuryl fluoride (SO_2_F_2_) and pentafluorooxosulfate (OSF_5_) anions was achieved. This was performed using fluorosulfonyl pentafluorooxosulfate (FSO_2_‐OSF_5_) a stable, high boiling liquid, obtained from SO_2_ and the pentafluorosulfur hypofluorite (FOSF_5_) prepared quantitatively in gram‐scale. Here, the gas free anion precursor of the emerging OSF_5_ chemistry and a dual‐SuFEx reagent for one‐pot synthesis of Sulfonyl fluoride (─SO_2_F) and Sulfurimidoyl Fluoride (─N═S(O)F_2_) is presented.

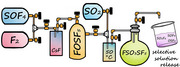

## Introduction

Extending the scope of biologically active fluorinated moieties has been a long indirect collaboration between inorganic and organic chemistry. One recent example is the investigation of the pentafluorosulfanyl (─SF_5_) group.^[^ ^1^, ^2^ ^]^ Indeed, from the first synthesis using elemental fluorine (F_2_), numerous synthetic improvements were reported up until recently, with the efficient TCICA/KF combination approach.^[^ ^3^ ^]^ This new accessibility has allowed pentafluorosulfanylation reactions and related (bio)physical applications to be extensively studied.^[^ ^4^, ^5^, ^6^, ^7^, ^8^, ^9^, ^10^, ^11^, ^12^, ^13^, ^14^, ^15^, ^16^ ^]^ For polyfluorinated R─S^VI^F_5_ systems, the currently investigated gap concerns group 16 linkers insertion (R─Y─S^VI^F_5_) similarly to the development of trifluoromethyl (─CF_3_) chemistry.^[^ ^17^, ^18^, ^19^ ^]^ Indeed, addition of oxygen (─OCF_3_),^[^ ^20^, ^21^ ^]^ sulfur (─SCF_3_),^[^ ^22^, ^23^, ^24^, ^25^, ^26^, ^27^ ^]^ or selenium (─SeCF_3_)^[^ ^28^, ^29^, ^30^ ^]^ bridges have allowed beneficial (bio)physical modulation of the latter moiety.

The strong electron withdrawing effect (*‐I*) of the pentafluorosulfanyl moiety can be a drawback for biology‐oriented applications.^[^ ^31^, ^32^ ^]^ Therefore, it is expected that the use of pentafluorooxosulfate (─OSF_5_) can positively modify those properties by reducing electronic effects while inducing flexibility and oxygen interactions. The modern pentafluorooxosulfanylation chemistry^[^ ^33^, ^34^ ^]^ relies on the generation of silver(I) pentafluorooxosulfate (Ag[OSF_5_])^[^ ^35^, ^36^ ^]^ and the cation exchange strategy,^[^ ^37^ ^]^ as utilized by Pitts^[^ ^38^ ^]^ and Qing^[^ ^39^ ^]^ with a nucleophilic approach. While this method greatly restricts experimental optimization (solvent and concentration) for reaction design, it also permits to avoid handling of the scarcely available thionyl tetrafluoride (SOF_4_).

**Figure 1:**
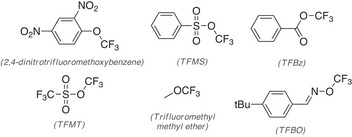
Nucleophilic trifluoromethoxy anion precursors.

The latter species was introduced by Moissan in 1901 and strongly studied by inorganic chemists for a century. More recently, Sharpless^[^ ^40^, ^41^, ^42^, ^43^, ^44^, ^45^, ^46^, ^47^, ^48^ ^]^ demonstrated its use as a versatile precursor for multidimensional SuFEx click chemistry.^[^ ^49^, ^50^, ^51^, ^52^, ^53^ ^]^ We newly re‐investigated this reagent^[^ ^54^ ^]^ and its synthesis allowing the handling of thionyl tetrafluoride in 100 mmol scale.^[^ ^37^ ^]^ We then studied OSF_5_ ion‐pairs and the influence of the cations in organic solvents. This methodology is complementary to the generation previously mentioned. Additionally, we questioned the design of a non‐gaseous reagent that would bring modularity to the pentafluorooxosulfate chemistry. We aimed to develop a reservoir able to release [OSF_5_]^−^ ions in a controlled manner. To meet these specifications, we considered the chemistry of trifluoromethoxy anionic precursors (Figure 1). Indeed, numerous well‐established reagents allow trifluoromethoxy anion release by reaction with a fluoride anion (X^+^F^−^) or base.^[^ ^55^, ^56^, ^57^, ^58^, ^59^, ^60^ ^]^

**Scheme 1:**
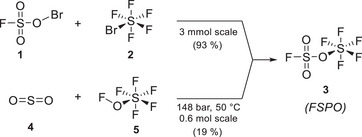
Reported synthesis of fluorosulfonyl pentafluorooxosulfate **3**.

We found in fluorosulfonyl pentafluorooxosulfate (**3**, FSO_3_SF_5_) a potential candidate for this chemistry. This “high boiling” (39.6 °C) and water stable^[^ ^61^ ^]^ liquid has been synthetically studied since 1963 but only partially characterised (IR, ^19^F NMR,^[^ ^62^, ^63^, ^64^ ^]^ and GED^[^ ^65^ ^]^). Interestingly, no applications were reported and its potential for modern (bio)organic chemistry was to be shown. Regarding its synthesis, two main pathways have been described (Scheme 1).

First, the reaction of fluorosulfate hypobromide (**1**, FSO_3_Br) with sulfur bromide pentafluoride (**2**, SF_5_Br) which allows access to the desired analogue **3** in high yield (Scheme 1).^[^ ^66^ ^]^ Unfortunately, this pathways requires the use of the poorly accessible S_2_O_6_F_2_ notably known for its potential hazardous synthesis ^[^ ^67^ ^]^ as well as the use of the BrSF_5_ **2** which is not commercially available.

The preparation of FSO_3_SF_5_ **3** was also reported from (OSF_5_)_2_ peroxide,^[^ ^68^ ^]^ fluorine‐fluorosulfate FOSO_2_F,^[^ ^69^ ^]^ and corona discharges of wet SF_6_.^[^ ^70^ ^]^ Additionally, the one‐pot oxidative pentafluorooxosulfanylation and fluorination of sulfur dioxide (**4**, SO_2_, 2 equiv., Scheme 1) was reported by Pass and Roberts in 1963 by thermal activation at 50 °C.^[^ ^61^ ^]^ In their report, the reaction was performed at medium temperature but high pressure (148 bars). In 1964, Ruff reported a similar reaction using light irradiation affording **3** in 30% yield.^[^ ^71^ ^]^ Since this route would allow the perfect atom economy reaction and SOF_4_ synthesis is now available in large scale in our laboratory, we decided to re‐investigate it for the preparation of **3**.

## Results and Discussion

**Scheme 2:**
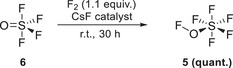
Synthesis of pentafluorosulfur hypofluorite **5**.

The synthesis of the required hypofluorite **5** was achieved by using a slight excess of *fluorine* (1.1 equiv.) and thionyl tetrafluoride **6** (Scheme 2).^[^ ^71^ ^]^ The preparation of **5** can also be achieved directly from SOF_2_.^[^ ^72^ ^]^ This catalysed reaction (using CsF allows fluorination of CsOSF_5_), led to the quantitative gram‐scale formation of pentafluorosulfur hypofluorite (**5**, 21.5 mmol, 3.5 g per batch) after fluorine removal. *! F_2_ and FOSF_5_ are strong oxidizers and react violently with organic material (see* *Supporting Information)*.

**Figure 2:**
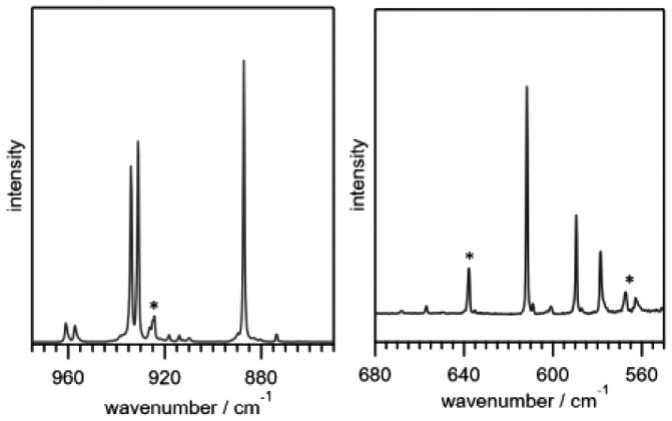
IR spectra at 5 K in neon matrix; Asterisk (*) indicate peaks originating from decomposition of **5** to SOF_4_ in the stainless‐steel line connected to the matrix chamber.

With this reagent in hand, we decided to update its structural and theoretical depiction. We first performed gas‐phase IR and the spectrum agreed with the literature data (Figure S10). To add further clarity in the IR characterization, we also recorded the IR spectrum by matrix‐isolation technique in a neon matrix at 5 K to reduce linewidth and overlaps (Figure 2).

The experimental bands were then assigned, supported by density functional theory (DFT) calculation, to *ν*_as_(OF) 961 cm^−1^, *ν*_as_(SF_eq_) 957 cm^−1^ and 934 cm^−1^,* ν*_as_(SF_ax_) 931 cm^−1^ and *ν*_s_(SF_eq_) 887 cm^−1^. At lower frequencies, we observed rocking (ρ) and scissoring (δ); ρ(SF_eq_) 612 cm^−1^, δ(SF_ax_) 590 cm^−1^ and δ (SF_eq_) 578 cm^−1^.

**Figure 3:**
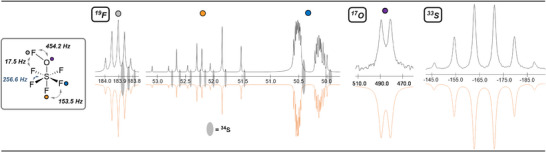
Multinuclear NMR Spectroscopic data of **5**: ^19^F (unscaled); ^17^O; ^33^S; Experimental spectra (top), simulation (bottom) performed with gNMR 5.0.

We then recorded (neat, −80 °C, uncorrected, see Supporting Information) the ^19^F, ^17^O, and ^33^S NMR of **5**. In the refined^[^ ^64^, ^73^ ^] 19^F NMR spectrum (Figure 3), the ─OSF_5_ group appears as an AB_4_ pattern, with a high order quintet (S–F_ax_) *δ* = 52.2 ppm and a high order doublet of doublet (S─F_eq_) at *δ* = 50.3 ppm with a measured ^2^J(F, F) of 153.5 Hz. At 183.9 ppm, we observe a first order quintet for the FOSF_5_ with a ^3^J(F, F_eq_) coupling of 17.5 Hz. A ^1^ΔF(^32^S─^33^S) isotope shift of −0.028 and −0.029 ppm was respectively observed for the equatorial and axial fluorines of ─OSF_5_. Regarding the hypohalite (F─O), the isotope shift is −0.014 ppm. In the ^17^O NMR spectrum, we observed a broad doublet at *δ* = 485.4 ppm with measured couplings ^1^J(O,F) of 454.2 Hz (Figure 3). The ^33^S NMR showed a broad sextet at −166.8 ppm with a ^1^J(S, F) of 256.6 Hz (Figure 3).

**Figure 4:**
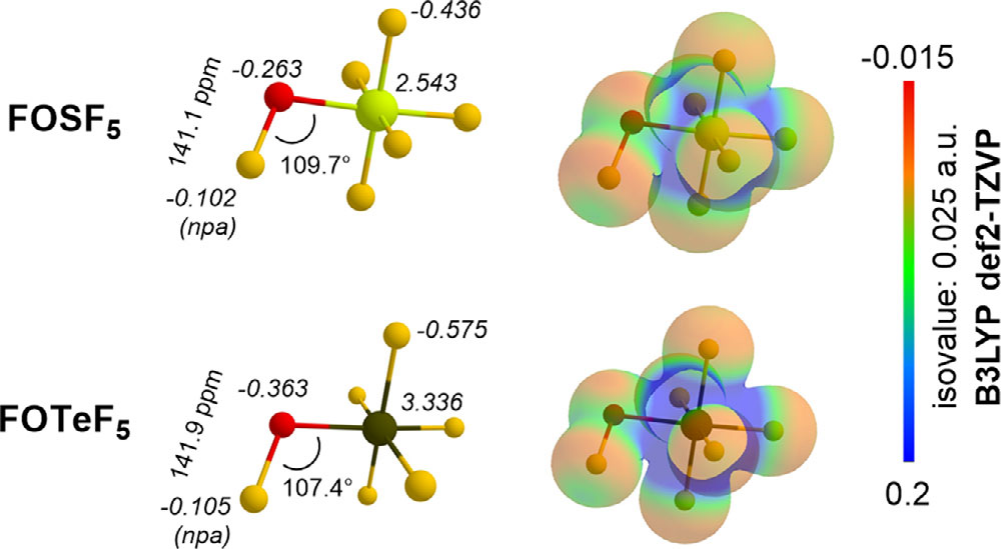
DFT calculations at the B3LYP/def2‐TZVP level, minimum structures of FOSF_5_ and FOTeF_5_, with italic values corresponding to the npa charges. Electrostatic potential mapped onto the electron density isosurface (0.025 a.u.).

We then performed DFT calculations on the hypofluorite **5** and its higher group 16 analogue (FOTeF_5_) at B3LYP/def2‐TZVP level using the ORCA 6 program package (Figure 4).

With the minimum structure of both species, we used the natural bond orbital (NBO 7.0) program to compare them. In both cases (FOYF_5_; Y = S, Te), the natural population analysis (NPA) charge showed a small negative one for both reactive fluorine atoms (F─OYF_5_) and that both chalcogen centers display large positive charges. Additional similarities can be observed in the comparable hypofluorite bond distances. The angles’ variations could be attributed to the steric hinderance induced by the center's radius. Overall, these results agree with the previous theoretical study of the electron‐withdrawing effect for OYF_5_ derivatives.^[^ ^74^ ^]^

**Scheme 3:**
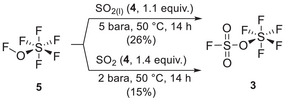
Preparation of fluorosulfonyl pentafluorooxosulfate **3**.

We then performed the reaction of **5** with sulfur dioxide **4** at different pressures. Firstly, the reaction was conducted at 5 bara with liquified SO_2_ at 50 °C to mimic the work of Pass (Scheme 3).

**Figure 5:**
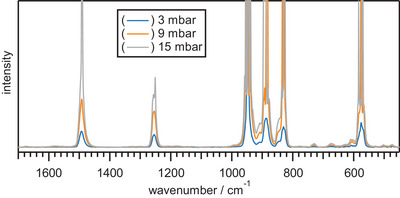
Gas‐phase IR spectra of **3**.

After 14 h, trap‐to‐trap distillation and further purification allowed the isolation of the desired FSO_3_SF_5_ in 26% yield as a clear liquid and was confirmed by IR spectroscopy (Figure 5).

The main subproducts observed are thionyl tetrafluoride **6** and sulfuryl fluoride (**7**, SO_2_F_2_). Compared to the initial study, no large amount of sulfur hexafluoride SF_6_ was formed and the yield was slightly higher than the reported 19%. Therefore, we envisaged that further reducing the pressure could allow a more selective synthesis. At 2 bara with 1.4 equiv. of SO_2_, we recovered **3** in 15% yield with fluorosulfonic acid (FSO_3_H) contamination. Further attempts have not permitted to increase the yield so far. With the FSPO in hand, we completed its missing characterization (neat ^19^F, ^17^O and ^33^S NMR). We first attempted to elucidate the solid‐state structure of FSO_3_SF_5_ by in‐situ crystallization on the diffractometer in a capillary tube at 166 K. Unfortunately, we observed glass transition that deeply complexified the preparation of a single crystal. Therefore, the elucidation is still ongoing in the laboratory.

**Figure 6:**
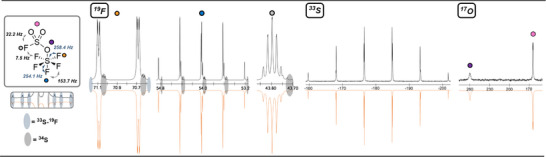
Multinuclear NMR Spectroscopic data of **3**: ^19^F (unscaled); ^17^O; ^33^S; Experimental spectra (top), simulation (bottom) performed with gNMR 5.0.

In the refined ^19^F NMR spectrum (Figure 6), the fluorine atoms of the ─OSF_5_ group appear as a high order doublet of doublet (S–F_eq_) *δ* = 70.9 ppm and a high order quintet (S─F_ax_) at *δ* = 54 ppm with the measured ^2^J(F,F) of 153.7 Hz. In the case of a strong electron withdrawing substituent (e.g. ─CF_3_^[^ ^75^ ^]^) on OYF_5_ species (Y = S, Se, Te), an inversion from AB_4_ to A_4_B pattern is noted for [OSF_5_]^–^. We also observed for the axial position a ^1^ΔF(^32^S─^33^S) isotope shift of −0.027 ppm, while the equatorial position is shifted by −0.028 ppm. For the pentafluorooxosulfate group of **3**, the average ^1^ΔF(^32^S─^33^S) is −0.0275 ppm. To our delight we also observed the rare quadrupole effect as small satellites lines of ^19^F─^33^S (0.7% abundancy). For the fluorosulfonyl (─SO_2_F) moiety, we observed a first‐order quintet at *δ* = 43.8 ppm and a spatial ^4^J(F, F) of 7.5 Hz with the ─SF_eq_. A ^1^ΔF(^32^S─^33^S) isotope shift of −0.025 ppm is observed.

Then, we managed to identify two distinct S─F coupling in ^33^S NMR at 30.7 MHz for the ─OSF_5_ moiety. The –OSF_5_ is seen at *δ* = −180.7 ppm as a doublet of quintet. The ^1^J(S, F_eq_) is 258.4 Hz and ^1^J(S, F_ax_) 254.1 Hz. Concerning ─SO_2_F, it displays a broad singlet at *δ* = −46.9 ppm (not depicted in Figure 6). The ^17^O NMR spectrum at 54.3 MHz showed a broad singlet for ─OSF_5_ and a doublet for ─SO_2_F respectively at *δ* = 259.7 and *δ* = 164.7 ppm with measured couplings ^2^J(O, F) of 32.2 Hz (Figure 6).

**Figure 7:**
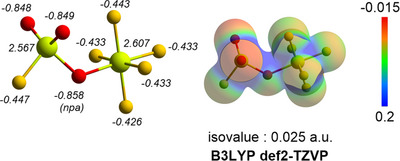
DFT calculation at the B3LYP/def2‐TZVP level, minimum structures of FSO_3_SF_5_, with italic values corresponding to the npa charges. Electrostatic potential mapped onto the electron density isosurface (0.025 a.u.).

To further study the FSO_3_SF_5_ species and the influence of the fluorosulfonyl group, we performed DFT calculation allowing the depiction of the ESP map and NBO analysis provided the NPA charges (Figure 7).

When compared to **5** (Figure 4, top), we observe a stronger electronic influence of the fluorosulfonyl on the –OSF_5_. Interestingly, **3** exhibits charge uniformity for each atom type. Both sulfurs display large positive charges and each oxygen bears most negative ones. Fluorine atoms charges show slight variations across the ─SF_5_ attributed to spatial/steric interactions.

**Scheme 4:**
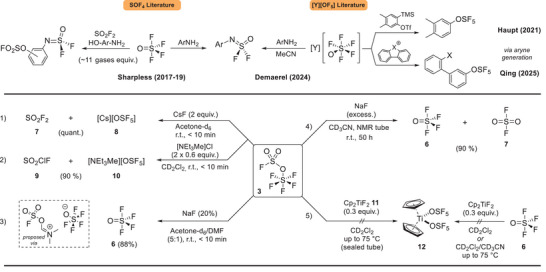
Reservoir ability of FSO_3_SF_5_: 1) Release of sulfuryl fluoride and caesium pentafluorooxosulfate anion; 2) Release of Sulfuryl chloride fluoride and ammonium pentafluorooxosulfate anion; 3) Selective release thionyl tetrafluoride; 4) Direct release of thionyl tetrafluoride and sulfuryl fluoride; 5) Unsuccessful preparation of novel transition metal bearing the pentafluorooxosulfate ligand.

Finally, inspired by the OCF_3_ chemistry and the initial work of Demaerel, De Borggraeve and Ismalaj, we hypothesized that **3** could act as a non‐gaseous, OSF_5_, SO_2_F_2_, and SOF_4_ reservoir (Scheme 4). First, the generation of Cs[OSF_5_] and SO_2_F_2_ was achieved in quantitative and rapid manner using an excess of CsF in deuterated acetone (Scheme 4.1). This reactivity was previously observed using CsF at 100 °C by Ruff in a solid‐gas interface reaction.^[^ ^71^ ^]^ Then, we decided to compare the formation of NEt_3_Me[OSF_5_] from FSO_3_SF_5_ and NEt_3_Me[Cl] versus Ag[OSF_5_] first published by our group^[^ ^37^ ^]^ and then by Qing^[^ ^39^ ^]^ who demonstrated its long term solid‐state stability at −30 °C. To our delight, we observed the formation of the desired ion‐pair **10** in 90% yield and sulfuryl chloride fluoride **9**, highlighting the versatility of **3** for the controlled formation of OSF_5_ anions. A small part (10%) was also converted to SO_2_F_2_/SOF_4_ (Scheme 4.2).

We then envisaged that using sodium fluoride (NaF) could trigger the decomposition of the supposedly instable NaOSF_5_ ion‐pair (Equation 1).

(1) $$\text{Na}\left[{\text{OSF}}_{5}\right]\to \text{NaF}+{\text{SOF}}_{4}$$

As the lattice energy is high and the solubility of NaF is low, the reaction would proceed at the solid‐liquid interface. After shaking the reaction medium, we first observed traces of SOF_4_. We then attempted the addition of dimethylformamide (DMF) to increase the solubility of the fluoride salt (Scheme 4.3). Almost immediately the decomposition of **3** and the release of SOF_4_ **6** in 88% yield was observed. We also formed 12% of an unknown OSF_5_ ion‐pair. Sulfuryl fluoride (33.2 ppm) was formed as traces with a major compound observed at 37.2 ppm. This is close to organic compounds bearing a fluorosulfate moiety or the [FSO_3_]^−^ anion. Therefore, we first envisaged the nucleophilic attack on the ─SO_2_F by the DMF affording a non‐stabilized OSF_5_ and a cationic species. The [OSF_5_]^−^ could then decompose in the desired SOF_4_ and the onium in a more stable [FSO_3_]^−^ anion. Therefore, a selective release of **6** was obtained and one could directly use the generated thionyl tetrafluoride in a one‐pot synthesis. We also observed that Cs_2_CO_3_ containing low amount of CsOH could generate the sulfuryl fluoride free formation of CsOSF_5_ with possibly FSO_3_Cs and CO_2_.

Using an excess of NaF (17 equiv. shaken in the NMR tube, Scheme 4.4) we observed the slow SOF_4_ and SO_2_F_2_ generation. This reaction was performed in a Young NMR tube; faster reaction rate should be obtained with proper stirring. We also managed to spot the previously unknown Na[OSF_5_] ion‐pair (up to 11% during the release, ^19^F NMR) probably stabilized by MeCN_x_•Na adducts as previously reported for silver salts.^[^ ^37^ ^]^

We also examined our reagent for the preparation of new complexes (Equation 2) avoiding the potential use of AgOSF_5_ (Equation 3).^[^ ^31^, ^32^ ^]^

(2) $$\left[M-F\right]+{\text{SOF}}_{4}\to \left[M-{\text{OSF}}_{5}\right]$$

(3) $$\left[M-\text{Cl}\right]+{\text{AgOSF}}_{5}\to \left[M-{\text{OSF}}_{5}\right]+\text{AgCl}$$

Indeed, the direct insertion of thionyl tetrafluoride in metal‐fluoride bond would allow the formation of transition‐metal bearing the rare pentafluorooxosulfate ligand. We then attempted to extend the scope of known transition metal starting from titanocene difluoride **11**. According to the literature and the related bond dissociation energies (BDE: Ti─F 569 versus Ti─O 662 kJ mol^−1^), the extrusion of SO_2_F_2_ and insertion of OSF_5_ could proceed. Surprisingly, the desired reactivity was not observed even at high temperature (75 °C) but **3** showed high stability (Scheme 4.5). Further discussion on the thermal stability of **3** can be found in the Supporting Information (DSC analysis, Figures S3–S6). We then envisaged that the S─O bond cleavage of **3** must disfavor the formation of **12** and therefore performed a control experiment using an excess of SOF_4_. Unfortunately, we also did not observe the insertion of SOF_4_ in the Ti─F bond in contrast to what was reported for Cu or Ni.

**Scheme 5:**
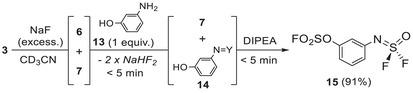
One‐pot gases release and successive formation of 3‐sulfurimidoylphenol **14** and 3‐sulfurimidoyl sulfonyl fluoride **15**, NMR yield. Y = S(O)F_2._

Finally, we believed that FSO_3_SF_5_ must have similar selectivity toward 3‐aminophenol **13** than SO_2_F_2_/SOF_4_ previously described by Sharpless (Scheme 4, top left). Therefore, we started investigating the dual‐SuFEx reactivity of **3** (Scheme 5). First, we attempted a one‐pot experiment that led to the formation of **15** in 25% but several subproducts (including ─NH─SO_2_F and ─N(─SO_2_F)_2_) and continuous scrambling were observed due to the phenol moiety.

To increase the selectivity, we slowly generated SOF_4_/SO_2_F_2_ from **3** using NaF. The addition of 3‐aminophenols **13** led to the formation of the sulfurimidoyl (─N═S(O)F_2_) **14** almost immediately with the formation of sodium bifluoride. We observed the described selectivity of aniline for SOF_4_ and no competition of the phenol. Then, N,N‐Diisopropylethylamine (DIPEA, 2 equiv.) was used for the formation of the sulfonyl fluoride moiety (─SO_2_F) affording the desired product **15** in 91% yield without the need of large excesses of **7** and **6**.

**Scheme 6:**
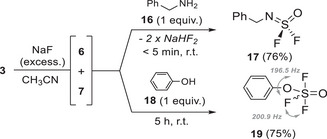
Selectivity of primary amine (top) and phenol (bottom) with SOF_4_/SO_2_F_2_ gases mixture.

Using benzylamine **16**, we confirmed the selectivity of primary amine with thionyl tetrafluoride **6** in the generated 1:1 gases mixture (Scheme 6, top). We observed the formation of the sulfurimidoyl **17** in 76% yield. Over‐reaction with SO_2_F_2_ **7** forming Bn‐NH‐SO_2_F was observed in 3% yield.

Finally, phenol **18** was added to the mixture of gases. To our delight, we observed the formation of phenoxythionyl trifluoride **19** in 75% yield without adding complementary base (i.e., DIPEA). In the refined ^19^F spectra,^[^ ^76^ ^]^ we observed a doublet of doublet for the equatorial fluorine with the measured ^2^J(F,F) of 200.9 and 196.5 Hz and two overlapped doublets for axial fluorines. We also observed for the equatorial position a ^1^ΔF(^32^S─^33^S) isotope shift of −0.030 ppm, while the axial positions are shifted by −0.021 and −0.020 ppm. These results also confirmed the higher electrophilic behavior and reactivity of the valuable thionyl tetrafluoride (Schemes 5 and 6). We also confirmed the higher reactivity of aniline/amine with **6**.

## Conclusion

To summarize, we have re‐investigated the fluorosulfonyl pentafluorooxosulfate (FSO_3_SF_5_) and the pentafluorosulfur hypofluorite (FOSF_5_) species. For both compounds, we provide the missing NMR characterization (^17^O and ^33^S) and thoroughly refined the known ^19^F NMR data.

With this work, we propose the first alternative to the reported OSF_5_ chemistry concerning the generation of key anions and gases by establishing FSO_3_SF_5_ (FSPO) as a convenient liquid reagent. Indeed, its use as reservoir for Sulfuryl Fluoride/Thionyl Tetrafluoride (SO_2_F_2_/SOF_4_) and pentafluorooxosulfate (^–^OSF_5_) anion was shown. Not only does this provide modularity for the emerging OSF_5_ chemistry, it allows the introduction of a new generation of reagents for multidimensional SuFEx click chemistry. We hope that with such reagent in hand, further improvements of the existent literature will be possible. So far, we found that the use of inexpensive NaF as hydrogen fluoride scavenger permits the base free (and glass etching free) formation of sulfurimidoyl fluoride. Further optimization and scale up are ongoing to access better yields. We believe that this class of practical reagents are necessary for the (bio)organic chemistry and for PFAS replacement research. We also actively investigate its use for the development of new distinct pentafluorooxo‐sulfanylation methodologies.

Finally, both the uses of FOSF_5_ as a direct transfer reagent and the synthesis and application of the corresponding (OSF_5_)_2_ peroxide are being developed by our group.

## Conflict of Interests

The authors declare no conflict of interest.

## Supporting information



Supporting Information

## Data Availability

The data that support the findings of this study are available in the Supporting Information of this article.
