# Supplementary material for: Assessing Fluorosulfonyl Pentafluorooxosulfate (FSO2–OSF5) Reservoir Capacity: Selective SOF4, SO2F2, and [OSF5]– Anion Release
Source: Angew Chem Int Ed Engl. 2025 Jul 30;64(36):e202510796. doi: 10.1002/anie.202510796 (PMC12402883; doi:10.1002/anie.202510796)
Supplement: Supplementary file 1 — Supporting Information [file ANIE-64-e202510796-s001.docx]

Table of Content

[General Information 1](#_Toc197938953)

[Experimental Section 2](#_Toc197938954)

[Experimental Spectra 9](#_Toc197938955)

[Computational Studies 21](#_Toc197938956)

[References 22](#_Toc197938957)

[Author Contributions 22](#_Toc197938958)

# General Information

Unless indicated otherwise, all experiments were carried out at room temperature and under inert conditions using standard Schlenk techniques and an argon atmosphere. Glassware was pre-dried before usage and greased with Triboflon III. Substances sensitive to hydrolysis and air were stored within a Sylatech glovebox (O_2_ < 0.05 ppm, H_2_O < 0.05 ppm) under an argon atmosphere. Amounts of gases were determined volumetrically based on the volume of the Schlenk line and the indicated pressure and were condensed at –196°C. Commercially available SO_2_ (Linde) was stored over CaH_2_, CsF was dried and activated as described in the experimental section. Dried solvents were used and stored over 3 Å molecular sieves. Gases were measured using a Nicolet iS5 FT-IR spectrometer that is directly connected to a Schlenk line. NMR spectra were recorded on a JEOL 400 MHz ECS or JEOL 400 MHz ECZ spectrometer. Graphics were created using Mestre Nova 14.2.^[1]^ NMR simulations were performed using gNMR 5.0. DSC analysis was performed using a Netzsch DSC 200 coupled with a TASC 414/3.

# Experimental Section

All the experiment involving elemental fluorine (F_2_) were conducted in a stainless steel Schlenk line depicted in Fig. S1. IR analysis and trap-to-trap purification were performed in a specific glass Schlenk line depicted in Fig. S2. All stainless-steel reactors were passivated using 2 bara of fluorine for 24h.


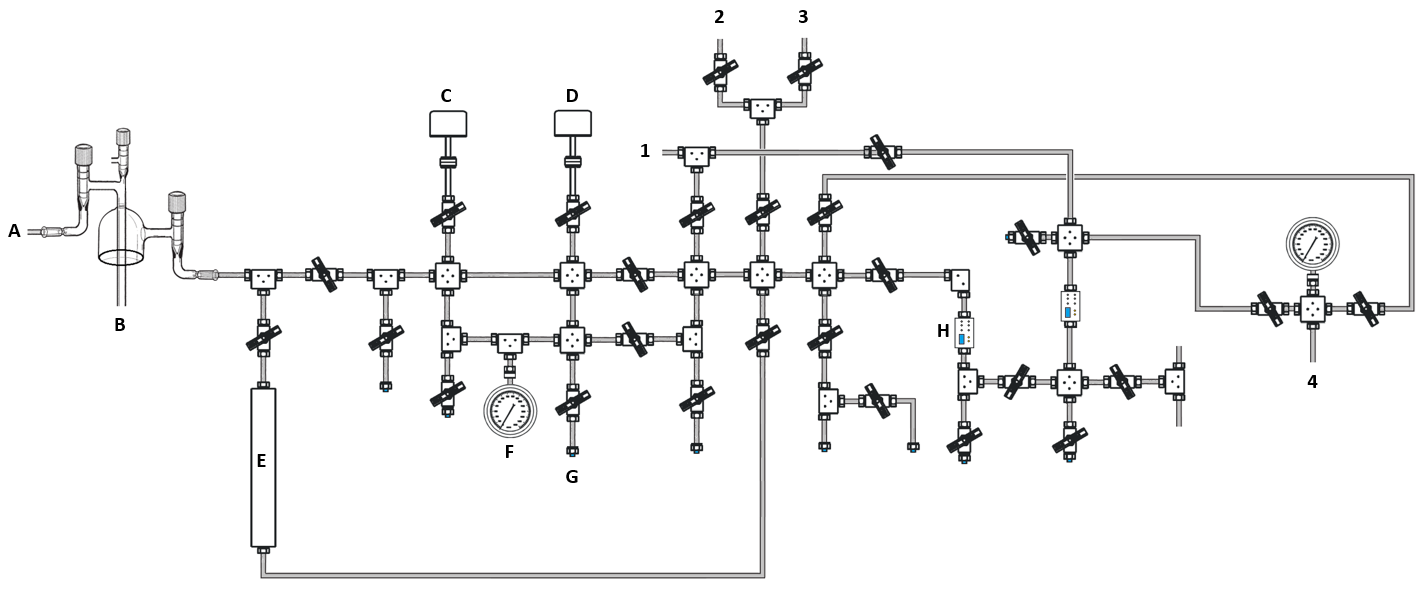


**Figure. S1**. Metal schlenk line; (A) To vacuum. (B) Vacuum primary trap. (C) Pressure gauge (<10 mbar). (D) Pressure gauge (<5 bar). (E) Soda lime. (F) Pressure gauge (9 bara). (G) Fitting female connectors. (H) Mass-flow controler. (1) Argon, (2) diluted F_2_ (20% N_2_), (3) OCF_2_ 100%, (4) F_2_ 100%.


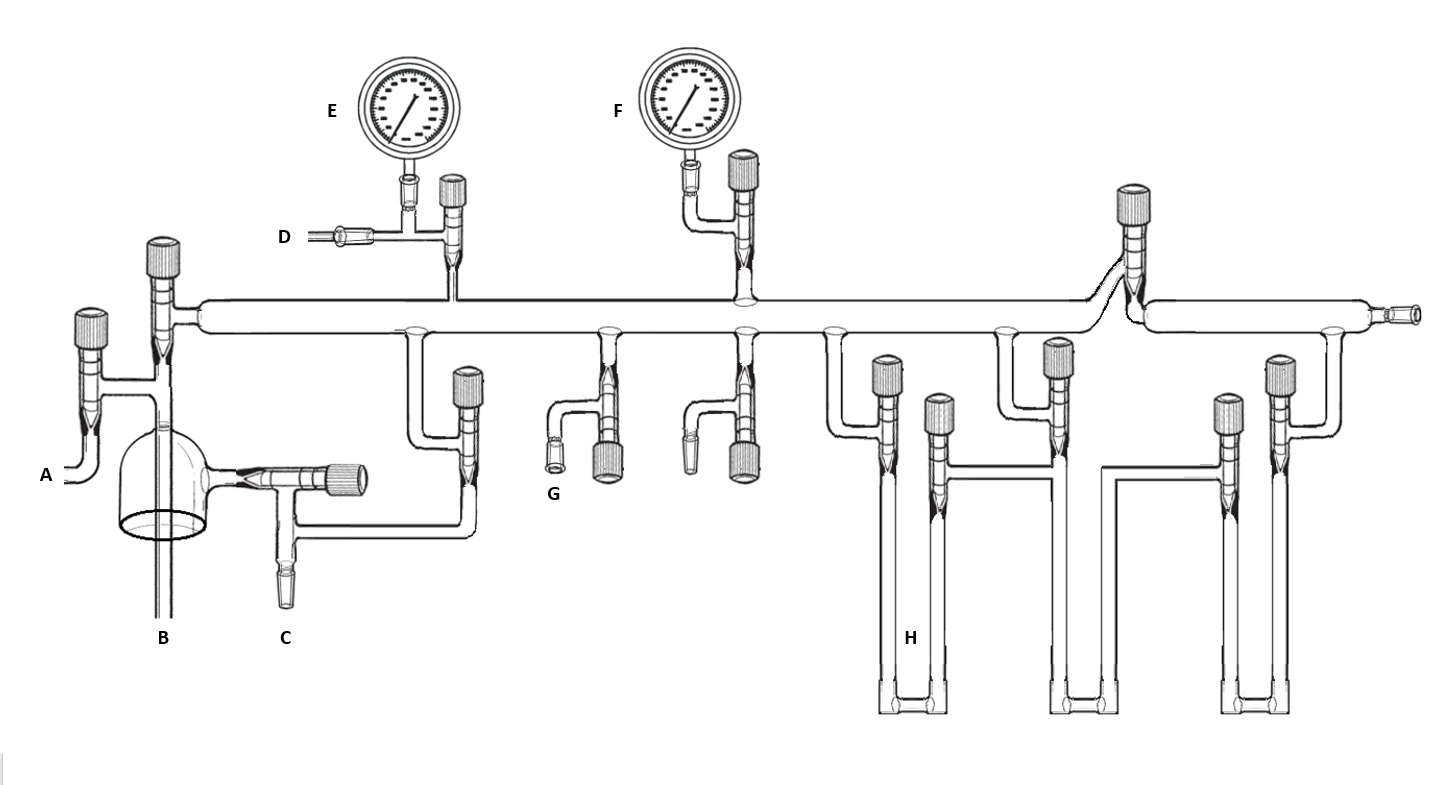


**Figure S2**. Distillation line & gas-phase IR; (A) To vacuum; (B) Vacuum primary trap; (C) Size 1 male connectors; (D) To IR; (E) Pressure gauge (100 mbar); (F) Pressure gauge (1 bar); (G) Size 1 female connectors; (H) U trap.

- **Activation of Caesium Fluoride (CsF)**

- For CsF catalyst (100g) used in a passivated stainless-steel reactor; caesium fluoride was dried at 110°C overnight under high vacuum and cleaned with elemental fluorine.

- For CsF used in the generation of [Cs][OSF_5_]; CsF (2 g, 13.2 mmol) was charged in a 25 mL Schenk tube and stirred overnight at 110°C.^[2]^ Then SO_2_ (15 mmol) was added and the medium was stirred until no consumption of SO_2_ was observed (via pressure drop). The excess of SO_2_ was pumped off leading to CsSO_2_F. Stirring the powder under high vacuum at room temperature then 100°C afforded 2g (100%) of the finely divided CsF.^[3]^

- **Synthesis of pentafluorosulfur hypofluorite FOSF_5_ (5)**

Pentafluorosulfur hypofluorite was prepared according to the literature.^[4]^ In a 660 mL reactor containing 100g of CsF was added 800 mbar (21,3 mmol) of SOF_4_ at room temperature followed by 900 mbar of F_2_ (23,9 mmol, 1.1 equiv.). The reaction medium was kept at room temperature and monitored by pressure drop indicating the formation of FOSF_5_. After 30h the pressure went from 1.7 bar to 900 mbar indicating full completion. The reactor was cooled to -196°C and the excess of fluorine pumped off. FOSF_5_ (100%) was then vacuum transferred to a passivated stainless-steel gas cylinder, no degradation was observed on storage at room temperature.

***Note: !*** We did not observe explosion during this work with inorganic material, but with **organic substances in further work, we report one violent explosion**. Therefore, **the handling must be done with extreme care** and with appropriate protection. To avoid potential additional risk with the condensed hypofluorite at −80°C we did not add a capillary tube, containing organic solvent, for shimming and referencing, the values are given uncorrected. For the ^19^F NMR, we observed a shift variation of Δδ_19F_(SOF_4_) = 3.08 ppm and Δδ_19F_(SO_2_F_2_) of 3.5 ppm. **^19^F NMR** (377 MHz, neat, −80°C): *δ* (ppm) = 183.9 (p, ^3^*J*(^19^F−^19^F)= 17.5 Hz, SO*F*); 52.2 (p, ^2^*J*(^19^F−^19^F)= 153.5 Hz, OSF_4_*F*); 50.3 (d, ^2^*J*(^19^F−^19^F)= 153.5 Hz, OS*F_4_*F); **^17^O NMR** (54 MHz, neat, −80°C): *δ* (ppm) = 485.4 (d, broad, ^1^*J*(^17^O−^19^F)= 454.2 Hz); **^33^S NMR** (31 MHz, neat, −80°C): *δ* (ppm)= −166.8 (sext, ^1^*J*(^33^S−^19^F)= 256.6 Hz).

- **Synthesis of Pentafluorosulfur Fluorosulfonate FSO_3_SF_5_ (3)**

Pentafluorosulfur fluorosulfonate was prepared from slightly modified literature procedure.^[5]^

*Gas-phase synthesis:*

In a passivated 80 mL stainless-steel autoclave was charged 1bar of SO_2_ (0.25 g, 3.9 mmol, 1.4 equiv.) followed by FOSF_5_ at -196°C (0.45 g, 2.7 mmol). The reactor was slowly warmed up to room temperature then heated to 50°C, the maximum pressure observed was 2 bara. After 15h, the reaction was cooled down to r.t. and the pressure dropped and indicated the end of the reaction. No residual SO_2_ or FOSF_5_ was observed in the crude by gas-phase IR. The desired product was isolated at -108°C via dynamic trap-to-trap distillation and quenching of FSO_3_H, pentafluorosulfur fluorosulfonate was obtained as a colourless liquid (15%, 90 mg, 0.4 mmol).

*High pressure synthesis*:

*Extreme care must be taken when performing high pressure fluorine chemistry see hereafter detailed safety considerations*

In a passivated 80 mL stainless-steel autoclave SO_2_ was condensed at -80°C (0.59 g, 9.2 mmol, 1.1 equiv.) followed by FOSF_5_ at -196°C (1.36 g, 8.4 mmol). The reactor was slowly warmed up to room temperature then heated to 50°C for 14h. As SO_2_ liquified (>3 bar), the maximum pressure observed was 5 bara. The desired product was first isolated at -108°C via dynamic trap-to-trap distillation with traces of fluorosulfuric acid (HSO_3_F). Pentafluorosulfur fluorosulfonate was obtained as a colourless liquid (26%, 500 mg, 2.2 mmol).

*Note*: Other traps contained large amounts of SO_2_F_2_ and SOF_4_ from the following equation : SO_2_ + FOSF_5_ 🡪 SO_2_F_2_ + SOF_4_ that can be separated and recovered for further experiments.

**IR**: 𝜈̃ (cm^-1^) = 1492 (m), 1254 (m), 947 (s), 886 (m), 830 (m); **^19^F NMR** (377 MHz, neat, external [D_6_]acetone, 21°C): *δ* (ppm) = 70.9 (d, ^2^*J*(^19^F−^19^F)= 153.7 Hz, OS*F_4_*F), 54.0 (p, ^2^*J*(^19^F−^19^F)= 153.7 Hz, OSF_4_*F*), 43.8 (p, ^4^*J*(^19^F−^19^F)= 7.5 Hz, SO_2_*F*); **^17^O NMR** (54 MHz, neat, external [D_6_]acetone, 21°C): *δ* (ppm) = 259.7 (s, broad, *O*SF_5_); 164.7 (d, ^2^*J*(^17^O−^19^F)= 32.2 Hz, S*O_2_*); **^33^S NMR** (31 MHz, neat, external [D_6_]acetone, 21°C): *δ* (ppm)=−46.9 (s, broad); −180.7 (dq, ^1^*J*(^33^S−^19^F_eq_)= 258.4 Hz, ^1^*J*(^33^S−^19^F_ax_)= 254.1 Hz).

*Observed NMR data of FSO_3_H:* **^19^F NMR** (377 MHz, neat, external [D_6_]acetone, 21°C): *δ* (ppm) = 45.5 (s); **^17^O NMR** (54 MHz, neat, external [D_6_]acetone, 21°C): *δ* (ppm) = 182.2 (s, broad, *O*H); 167.6 (broad, S*O_2_*).

- **Safety and stability considerations (DSC)**

Fluorine (F_2_) and FOSF_5_ are strong oxidizers. Both can (violently) react with organic material, releasing HF (exothermically). Thionyl tetrafluoride (SOF_4_) can also be hydrolysed, releasing HF. Handling must be done with great care in a well-ventilated fume hood with appropriate protection gears. Passivated (with fluorine) stainless steel line and metal/PFA reactors can be used to perform experiments. SOF_4_ can be used with dried glassware. Slow reaction occurs between SOF_4_ and the glassware overtime to generate SO_2_F_2_ and HF leading to glass etching.

Pentafluorosulfur hypofluorite (FOSF_5_) can be stored in stainless steel containers at room temperature without observing degradation. High purity can be obtained by performing trap-to-trap distillation. Irreversible decomposition occurs at 210°C with formation of SF_6_ and O_2_.^[6]^ Reaction with KOH (6 equivalents) leads to the formation of ½O_2_ + 5KF, 3H_2_O and KSO_3_F.^[7]^ No direct hydrolysis has been reported. Related experiment has been performed with pentafluorosulfur hypochlorite (ClOSF_5_) that reacts with H_2_O to give ClOH + HOSF_5_.^[8]^ The acid decomposes at –60°C releasing HF and SOF_4_. It is expected that the decomposition channel of FOSF_5_ with water occurs as follows:

H_2_O + FOSF_5_ **→** FOH + HOSF_5_

HOSF_5_ **→** SOF_4_ + HF

H_2_O + SOF_4_ **→** SO_2_F_2_ + 2HF

Concerning fluorosulfonyl pentafluorooxosulfate (FSO_3_SF_5_), we did not observe decomposition in solution up to 75°C (MeCN, DCM). Gas-phase synthesis was reported also up to 70°C. Decomposition in solid-gas experiment was reported at 100°C in presence of CsF. The water/acid stability was first described by Pass for purification of FSO_3_SF_5_ from SO_3_. The mixture was transferred in water and boiled at 100°C forming H_2_SO_4_ and distilled to afford the desired product.^[5]^

We also observed good stability of FSO_3_SF_5_ with acid. Indeed, isolation of FSO_3_H:FSO_3_SF_5_ (1:1) and storage in glassware showed decomposition of the fluorosulfonic acid with glassware but no decomposition of the fluorosulfonyl pentafluorooxosulfate. The same was observed with a similar mixture in MeCN or DCM.

We also studied the thermal stability of FSO_3_SF_5_ by performing DSC. As the reagent is a low boiling liquid (39.6°C), we prepared a flat bottom evacuated ampule containing 1 mg of the product as well as an empty reference (Figure S3) and placed them in the DSC chamber .

| *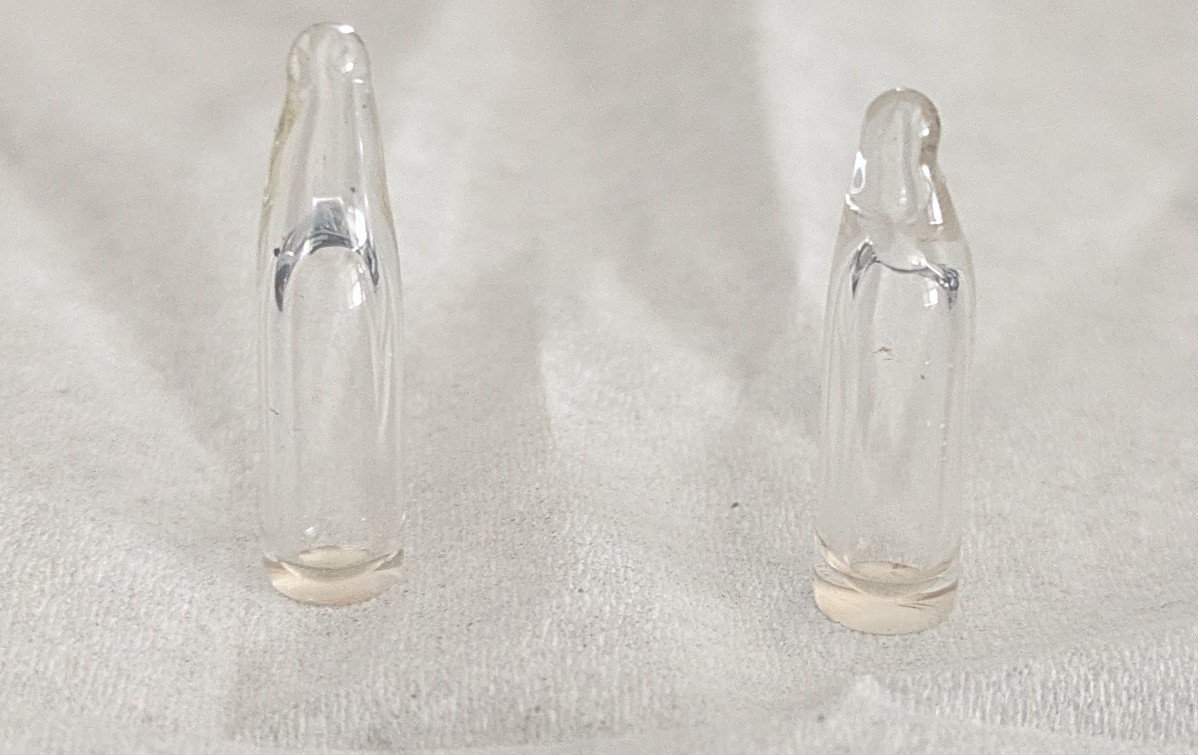* |  |
| --- | --- |
| **Figure S3.** Glass ampoule for DSC analysis of low boiling species. | |

The DSC sequence was conducted as follows. The sample was heated from 30 to 300°C at 3°C/min. The temperature was stabilized 15 min and slowly decreased up to 30°C. This was repeated a second time immediately (Figure S4). In this first experiment, only one isolating lid was used due to the experimental setup. Shortening the same glass ampule (see figure 6) and running the experiment with a second lid afforded refined spectra (Figure S5, S6).

**Figure S4.** DSC analysis of FSO_3_SF_5_ up to 300°C (using one lid for thermal isolation). ***** : FSO_3_SF_5_ b.p. **^■^** : water-surface release

**Figure S5.** DSC analysis of FSO_3_SF_5_ up to 400°C (using two lids for thermal isolation). ***** : FSO_3_SF_5_ b.p.; ^Δ^: Endothermic event.

We observed in both measurements the boiling point of 39.6°C (*****) as described in the literature. No obvious gas-phase decomposition was spotted in the first run (Figure S4). It was observed that a significant quantity of water was removed from the metal surface during the initial trial (see **^■^**). No damage of the glass ampule was observed during this experiment due to the low loading (1 mg). We then decided to run the same sample up to 400°C (Figure S5). Interestingly, still no obvious decomposition was observed, suggesting a high thermal stability of the compound in the gas phase. We saw the beginning of an endothermic event (Δ) at 330°C. To confirm the possible decomposition at higher temperature (> 400°C) we then used the same batch for a third run up to 500°C (Figure S6).

**Figure S6.** DSC analysis of FSO_3_SF_5_ up to 500 °C (left, using two lids for thermal isolation). ***** : FSO_3_SF_5_ b.p.; **^▼^**: Endothermic event provoking decomposition of the compound as proved by complementary DSC analysis (25 to 100°C); **^▲^** : minimum temperature reached by the apparatus was 51°C. Right: picture of the glass ampoule after the analysis, observation of glass etching from the decomposition of **3** for the first time.

To our delight, we finally observed decomposition (**^▼^**) of FSO_3_SF_5_ after performing analysis up to 500°C (maximum temperature). In both heating phases, an endothermic event is observed. The second endothermic event up to 500°C suggests only a partial decomposition of **3** in the first part of the experiment. Unfortunately, the apparatus did not manage to cool down to 30°C (**^▲^**) to observe the boiling point of FSO_3_SF_5_ (*****). Also, decomposition was confirmed by the glass etching, no clear decomposition temperature was observed (between 400 to 500°C). A last run (25 to 100°C) was performed and confirmed the disappearance of the starting material as no product with a boiling point of 39.6°C was observed. These experiments evidenced the unexpected high-thermal stability of **3** in the gas phase.

- **General procedure for the release of pentafluorooxosulfate anion or gases**

In a dried Young NMR tube was added the nucleophile. The tube was then evacuated, and the desired solvent added followed by the addition of FSO_3_SF_5_ via vacuum transfer. **3** can also be added using a syringe (density: 1.867).^[9]^

**Eq.1:** Following the general procedure, CsF (76 mg, 0.5 mmol, 2 equiv.) was added to a Young NMR tube. The tube was then evacuated before addition of deuterated acetone (0.5 ml) and FSO_3_SF_5_ (20 mbar, 0.25 mmol) via vacuum transfer at -196°C.The reaction medium was warmed up to room temperature, shaken vigorously and monitored by ^19^F NMR. NMR data agreed with the literature and SO_2_F_2_ and CsOSF_5_ were obtained quantitatively.^[10]^ **^19^F NMR** (377 MHz, [D_6_]acetone, 21 °C): *δ* (ppm) = 132.4 (quint, ^2^*J* = 159.2 Hz, F_ax_, 1F), 90.3 (d, ^2^*J* = 159.2Hz, F_eq_, 4F), 33.2 (s, SO_2_F_2_, 2F).

**Eq.2:**  Following the general procedure [NEt_3_Me]Cl (23 mg, 0.15 mmol, 0.6 equiv.) was added to a Young NMR tube. The tube was evacuated before addition of deuterated dichloromethane (0.5 ml) and FSO_3_SF_5_ (20 mbar, 0.25 mmol) via vacuum transfer at -196°C. The tube was warmed up to room temperature, shaken vigorously and monitored by ^19^F NMR. We observed the expected half conversion in SO_2_ClF and the ammonium OSF_5_ in less than five minutes plus the unexpected formation of SOF_4_, SO_2_F_2_. Then, [NEt_3_Me]Cl (23 mg, 0.15 mmol, 0.6 equiv.) was added in a glovebox at room temperature and the full consumption of **3** was observed. SO_2_ClF and [NEt_3_Me]OSF_5_ were obtained in 90% yield and SOF_4_/SO_2_F_2_ in 10% yield. NMR data agreed with the litterature.^[10,11]^ **^19^F NMR** (377 MHz, CD_2_Cl_2_, 21 °C): *δ* (ppm) = 133.0 (quint, ^2^*J* = 162.0 Hz, F_ax_, 1F), 98.5 (s, SO_2_ClF, 1F), 90.9 (d, ^2^*J* = 162.0 Hz, F_eq_, 4F), 79.7 (s, SOF_4_, 4F), 33.3 (s, SO_2_F_2_, 2F).

**Eq.3:** Following the general procedure, NaF (2 mg, 0.05 mmol, 0.2 equiv.) was added to a Young NMR tube. The tube was evacuated before addition of deuterated acetone (0.5 ml) and FSO_3_SF_5_ (20 mbar, 0.25 mmol) via vacuum transfer at -196°C. The tube was warmed up to room temperature, shaken vigorously and monitored by ^19^F NMR. We only observed low conversion to SOF_4_/SO_2_F_2_. 0.1 mL of DMF was added at room temperature under an argon atmosphere and almost immediate NMR analysis showed the full conversion of **3** and thionyl tetrafluoride was obtained in 88%. An unknown OSF_5_ anion was obtained in 12% yield. NMR data agreed with the literature.^[10]^ **^19^F NMR** (377 MHz, CD_2_Cl_2_, 21°C): *δ* (ppm) = 132.6 (quint, ^2^*J* = 159.6 Hz, F_ax_, 1F), 90.7 (d, ^2^*J* = 159.6Hz, F_eq_, 4F), 79.7 (s, SOF_4_, 4F), 37.2 (s, SO_2_F•DMF, 2F), 33.3 (s, *traces* SO_2_F_2_, 2F).

**Eq.4:** Following the general procedure, an excess of NaF (80 mg, 1.9 mmol, 17 equiv.) was added to a Young NMR tube. The tube was evacuated before addition of deuterated acetonitrile (0.5 ml) and FSO_3_SF_5_ (10 mbar, 0.12 mmol) via vacuum transfer at -196°C. The tube was warmed up to room temperature, shaken vigorously and monitored by ^19^F NMR. During the gas release we managed to observe up to 10% of NaOSF_5_. **^19^F NMR** (377 MHz, CD_3_CN, 21°C): *δ* (ppm) = 132.3 (quint, ^2^*J* = 159.1 Hz, F_ax_), 90.2 (d, ^2^*J* = 159.1Hz, F_eq_). After 50h, we obtained 90% of the desired SOF_4_ and SO_2_F_2_ NMR data agreed with the literature.^[10]^ **^19^F NMR** (377 MHz, CD_3_CN, 21°C): *δ* (ppm) = 79.1 (s, SOF_4_), 33.2 (s, SO_2_F_2_).

**Eq.5:** Following the general procedure, Cp_2_TiF_2_ (4.5 mg, 0.3 equiv.) was added to a Young NMR tube. The tube was evacuated before addition of deuterated dichloromethane (0.5 ml) and FSO_3_SF_5_ (5 mbar, 0.06 mmol) via vacuum transfer at -196°C. The similar reaction with Cp_2_TiF_2_ (13.7 mg), SOF_4_ (15 mbar, 0.19 mmol) and 0.5 mL of CD_2_Cl_2_ or 1 mL CD_2_Cl_2_/CD_3_CN (1:1) was performed. The tube was warmed up to room temperature, shaken vigorously and monitored by ^19^F NMR. For both experiments, no conversion was observed, the temperature was slowly increased up to 70°C without inducing conversion or decomposition.

**Eq.6:** Using the generated SOF_4_/SO_2_F_2_ from Eq.4, 3-aminophenol (12.5 mg, 0.11 mmol, 1 equiv.) was added to the Young NMR tube at room temperature. The tube was shaken vigorously and quickly monitored by ^19^F NMR. The reaction medium immediately turned light yellow and the ^19^F analysis showed the full conversion of SOF_4_ and residual NaOSF_5_ in the desired sulfurymidoyl **14** and SO_2_F_2_. Then, DIPEA (38 µL, 0.22 mmol, 2 equiv.) and fluorobenzene as internal reference for yield determination were added under argon at room temperature. 3-((Difluoro(oxo)-λ6-sulfanylidene)amino)phenyl sulfurofluoridate **15** was obtained in 91% yield and NMR data agree with the literature.^[12,13]^ **^19^F NMR** (377 MHz, [D_6_]acetone, 21°C): *δ* (ppm) = 44.8 (s, 2F, RN=S(O)**F_2_**), 36.8 (s, 1F; RSO_2_**F**).

**Eq.7:** Following the general procedure, an excess of NaF (85 mg, 2.0 mmol, 11.2 equiv.) was added to a Young NMR tube. The tube was evacuated before addition of acetonitrile (0.5 ml) and FSO_3_SF_5_ (15 mbar, 0.18 mmol) via vacuum transfer at
–196°C. The tube was warmed up to room temperature, shaken vigorously and monitored by ^19^F NMR. After complete gas release (3 days), the tube was frozen, quickly opened under argon flux and fluorobenzene as internal reference followed by freshly distilled benzylamine (20 µL, 0.18 mmol) were added to the NMR tube. Benzylsulfurimidoyl difluoride **17** was obtained in 76% yield, and we observed 3% of benzylsulfamoyl fluoride (Bn-NH-SO_2_F). NMR data agree with the literature.^[14]^ **^19^F NMR** (377 MHz, MeCN, 21°C): *δ* (ppm) = 48.7 (s, 1F, Bn-NH-SO_2_F), 45.3 (s, 2F, RN=S(O)F_2_), 32.6 (s, 2F; SO_2_F_2_). *Note:* we observed loss of gases by ^19^F NMR attributed to the experimental procedure (opening of Young NMR tube) possibly enhanced by the high concentration of gases (~0.4M per gas), lowering the yield. We confirmed this observation by repeating it without cooling, affording **17** in 39% yield. This also agrees with the maximum concentration of SOF_4_ determined by Sharpless (0.26 M at r.t. in saturated MeCN).^[12]^ Using vacuum transfer or addition through a septum would avoid this issue.

**Eq.8:** Following the general procedure, an excess of NaF (90 mg, 2.1 mmol, 7 equiv.) was added to a Young NMR tube. The tube was evacuated before addition of deuterated acetonitrile (0.5 ml) and FSO_3_SF_5_ (25 mbar, 0.30 mmol) via vacuum transfer at -196°C. The tube was warmed up to room temperature, shaken vigorously and monitored by ^19^F NMR. After complete gas release (3 days), the tube was frozen at -196°C, quickly opened under argon flux and fluorobenzene as internal reference followed by phenol (28.2 mg, 0.3 mmol) were added to the NMR tube. The tube was shaken for 5h. ((Trifluorosulfinyl)oxy)benzene **19** was obtained in 75% yield. NMR data agree with the literature.^[15]^ **^19^F NMR** (377 MHz, MeCN, 21°C): *δ* (ppm) = 85.54* (d, PhOS(O)F_2(ax)_F_eq_, 2F) 65.55 (dd, PhOS(O)F_2_F_eq_ ^2^*J* = 200.9, 196.5 Hz, F_eq_, 1F)). *: Both doublets overlap, the ^2^J can be found from the F_eq_.

# Experimental Spectra

| 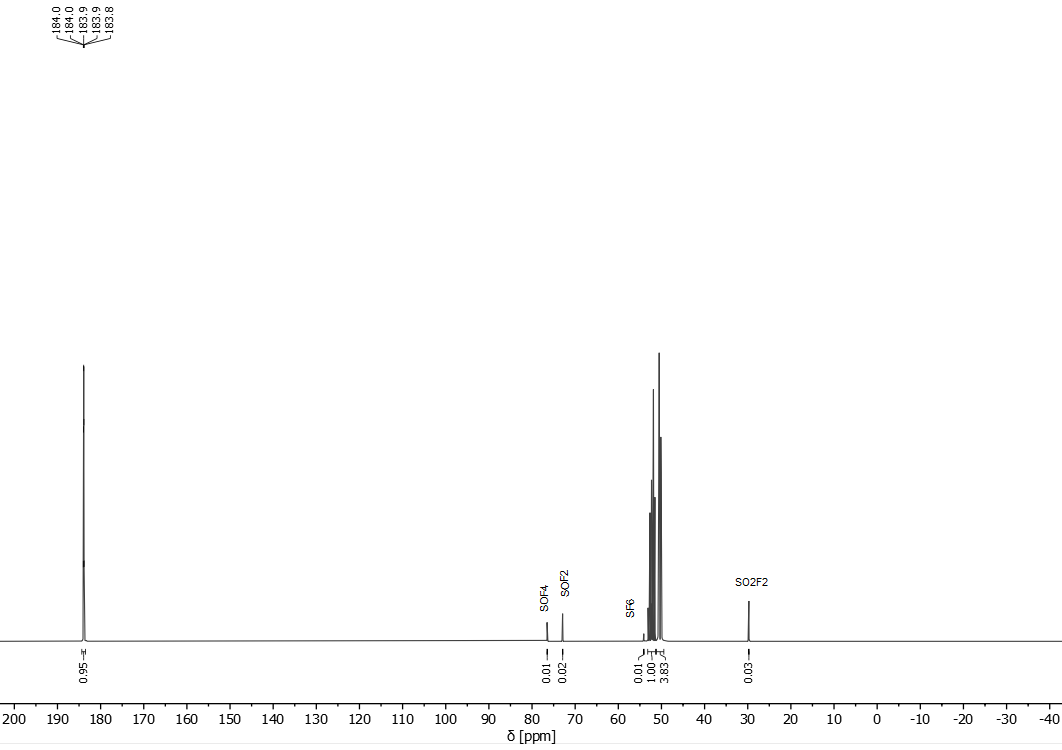 |
| --- |
| **Figure S7.A** Experimental ^19^F NMR spectrum (377 MHz, neat, −80°C) of FOSF_5_ (**5**). Observed decomposition and residual impurity from fluorine are explicited. |
| 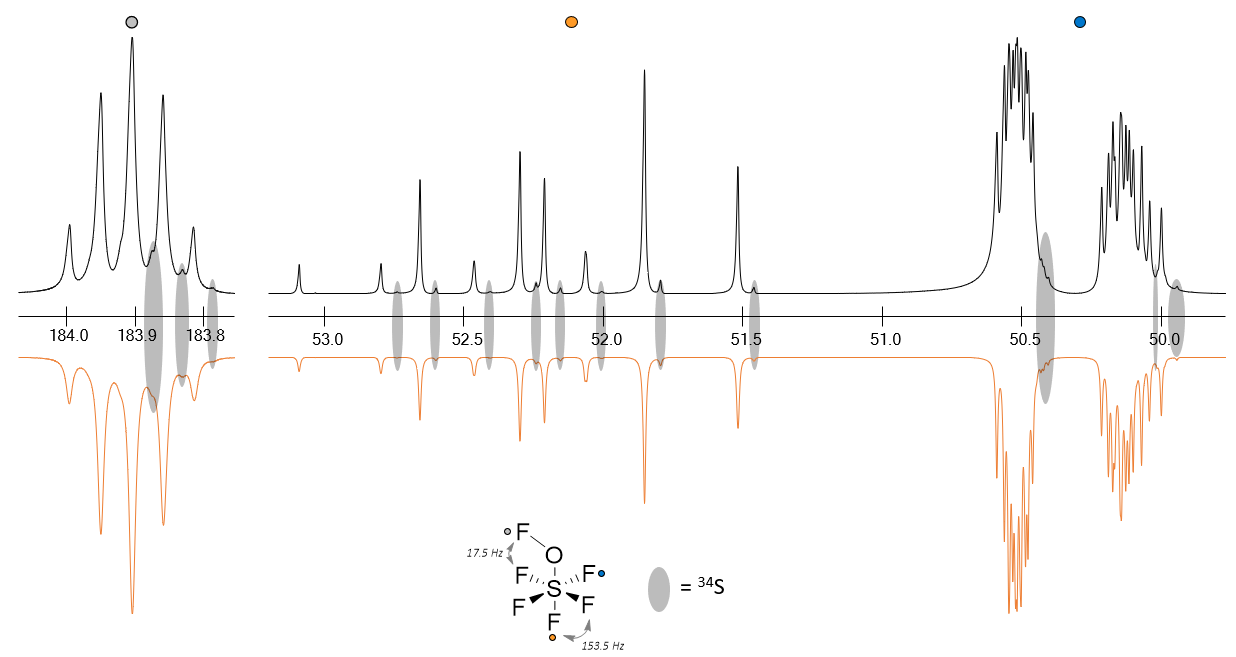 |
| **Figure S7.B** Experimental and simulated ^19^F NMR spectra (377 MHz, neat, −80°C) of FOSF_5_ (**5**) |

| 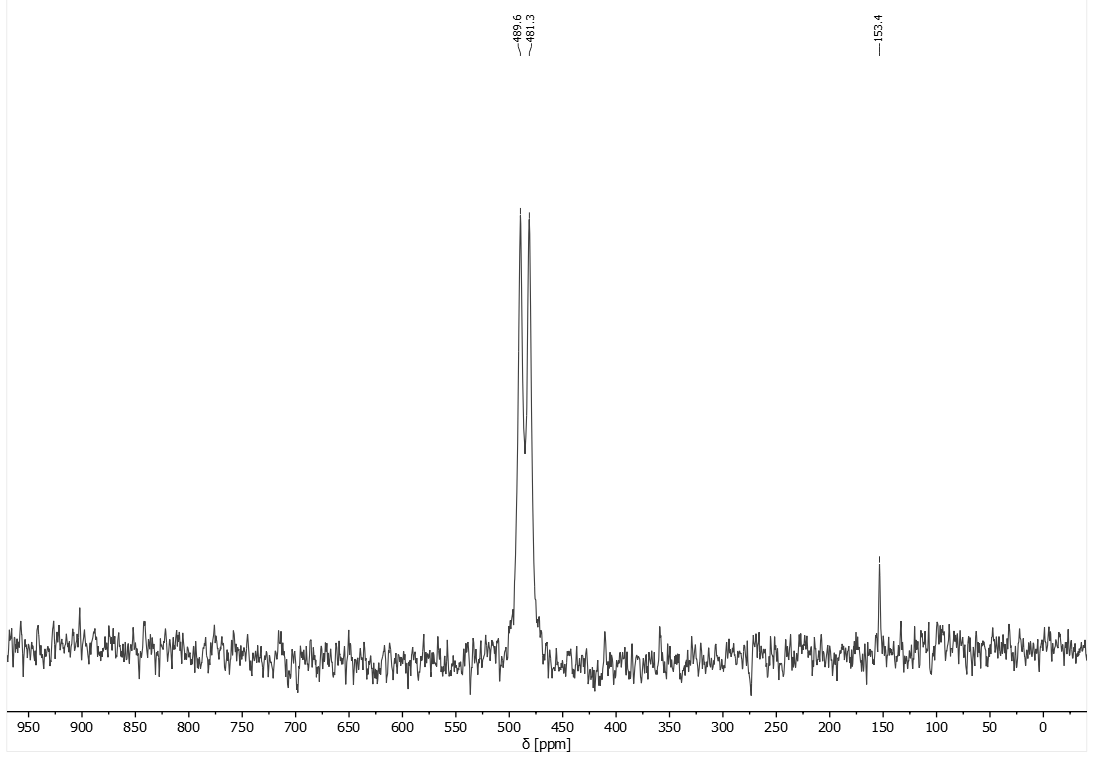 |
| --- |
| **Figure S8.A** Experimental ^17^O NMR spectrum (54 MHz, neat, −80°C) of FOSF_5_ (**5**). SO_2_F_2_ observed at 153.4 ppm. |
| **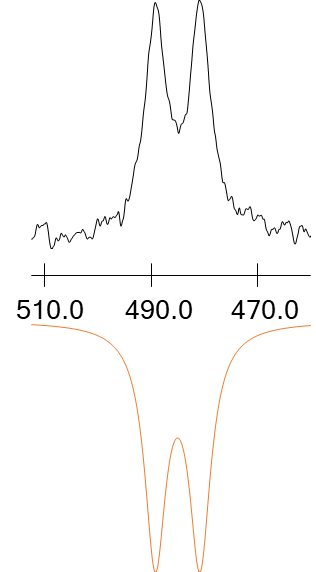** |
| **Figure S8.B** Experimental and simulated ^17^O NMR spectra (54 MHz, neat, −80°C) of FOSF_5_ (**5**) |

| 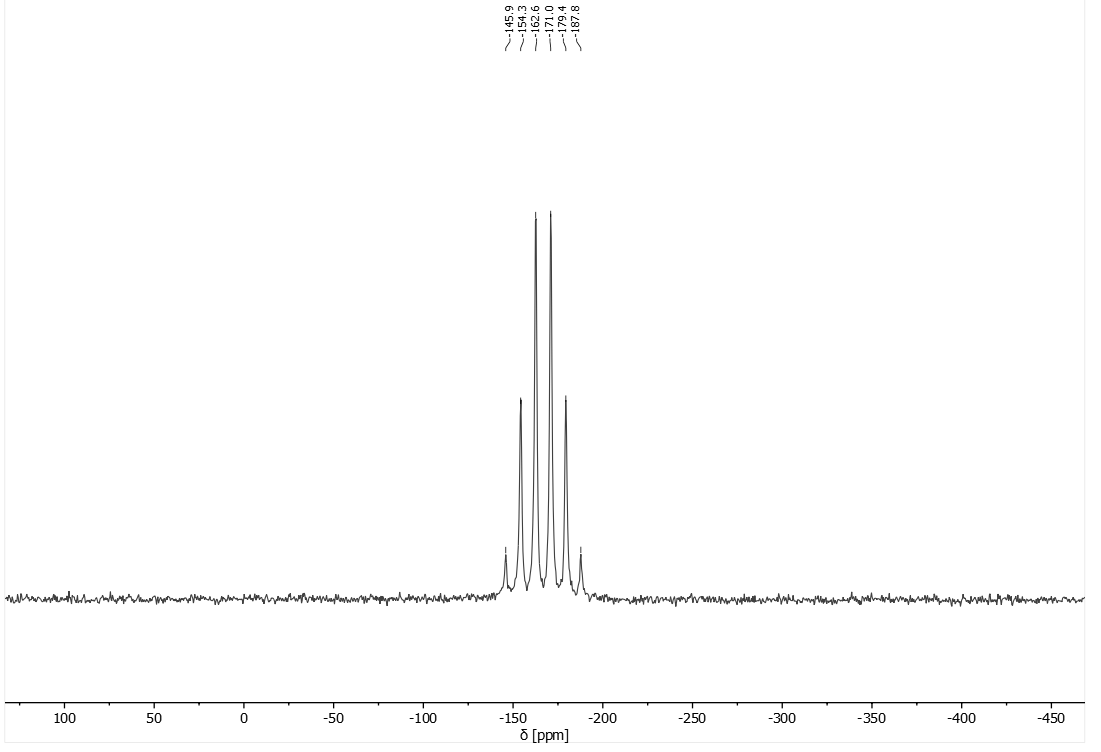 |
| --- |
| **Figure S9.A** Experimental ^33^S NMR spectrum (31 MHz, neat, −80°C) of FOSF_5_ (**5**) |
| **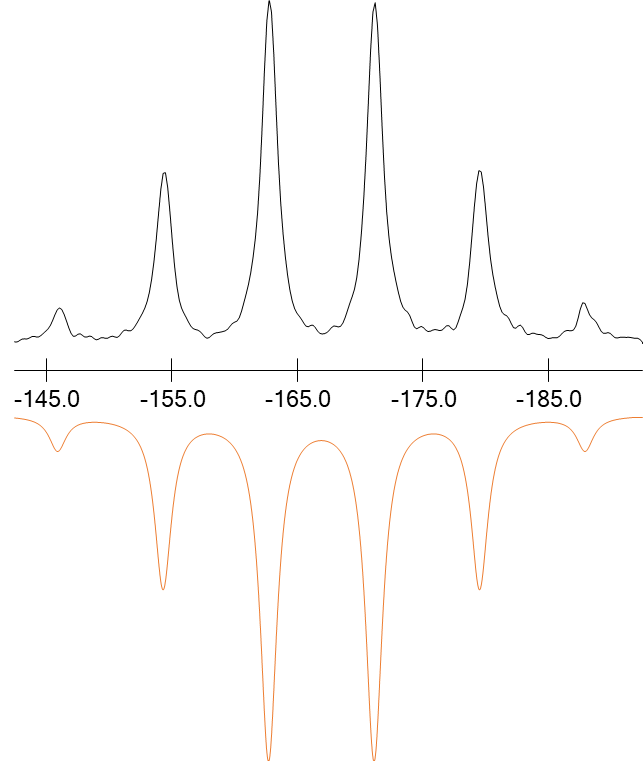** |
| **Figure S9.B** Experimental and simulated ^33^S NMR spectra (31 MHz, neat, −80°C) of FOSF_5_ (**5**) |

| 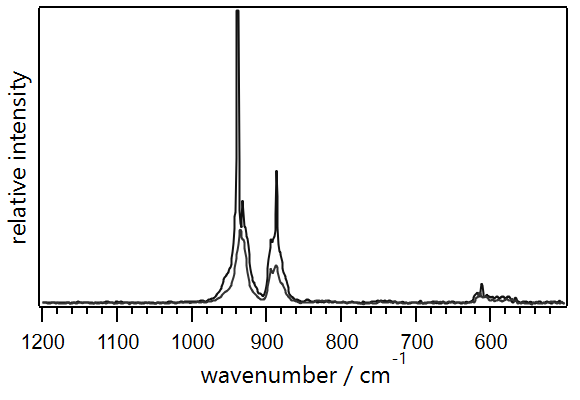 |
| --- |
| **Figure S10.A** Experimental IR spectra of FOSF_5_ (**5**) |
| 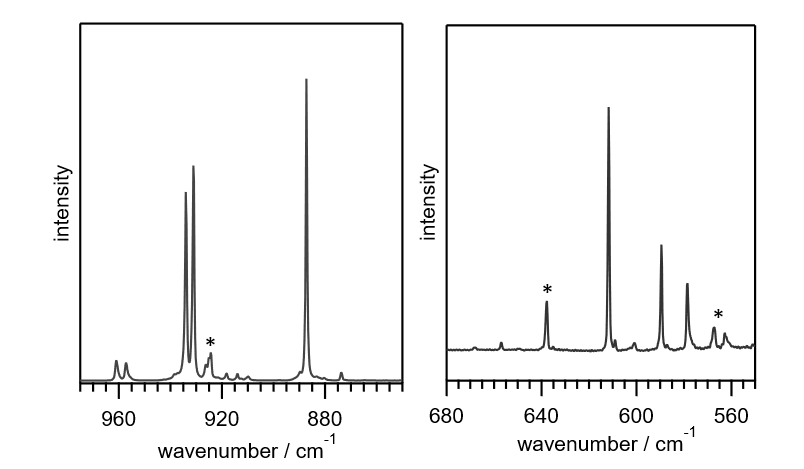 |
| **Figure S10.B** Experimental matrix IR spectra of FOSF_5_ (**5**) Asterisk (*) indicate peaks from SOF_4_ and from decomposition of **5** in the matrix line. |

| 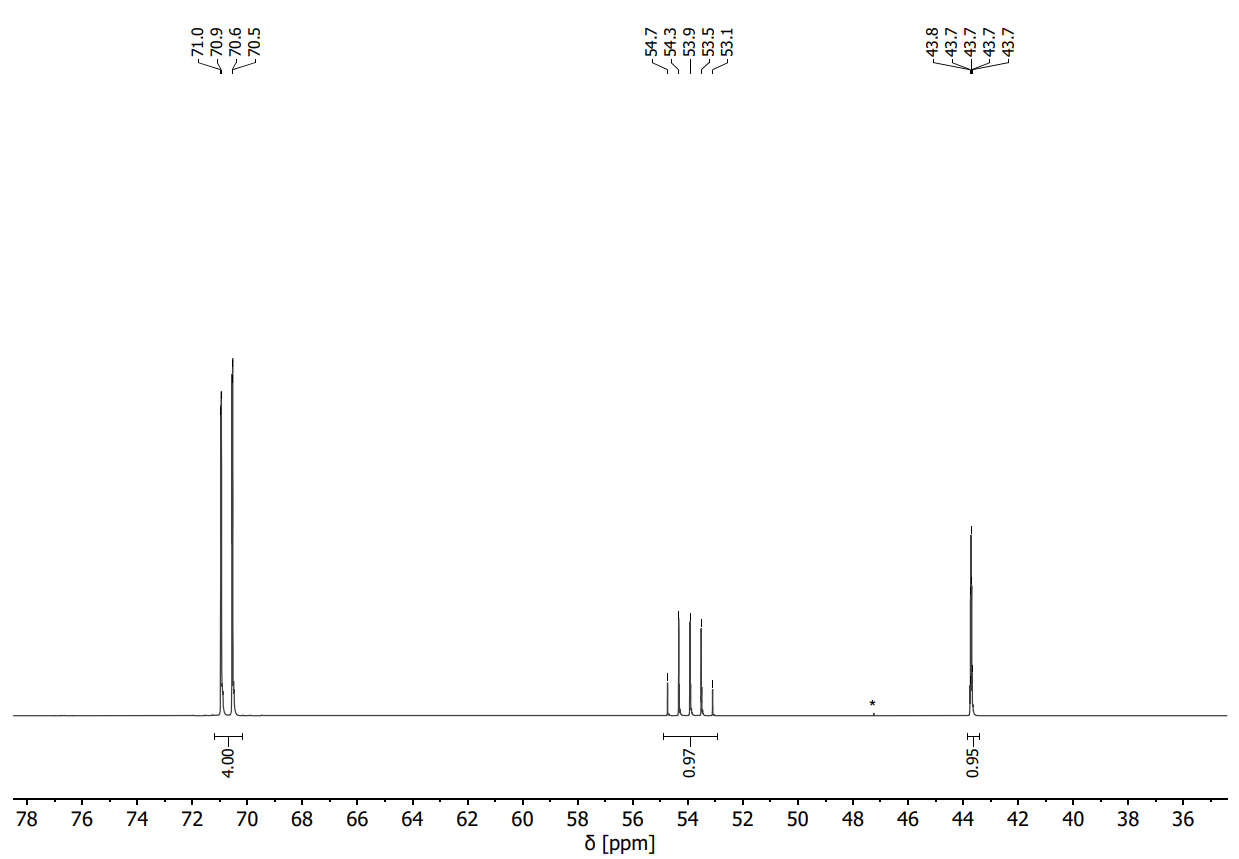 |
| --- |
| **Figure S11.A** Experimental ^19^F NMR spectrum (377 MHz, neat, r.t.) of FSO_3_SF_5_ (**3**). * : FSO_3_H |
| **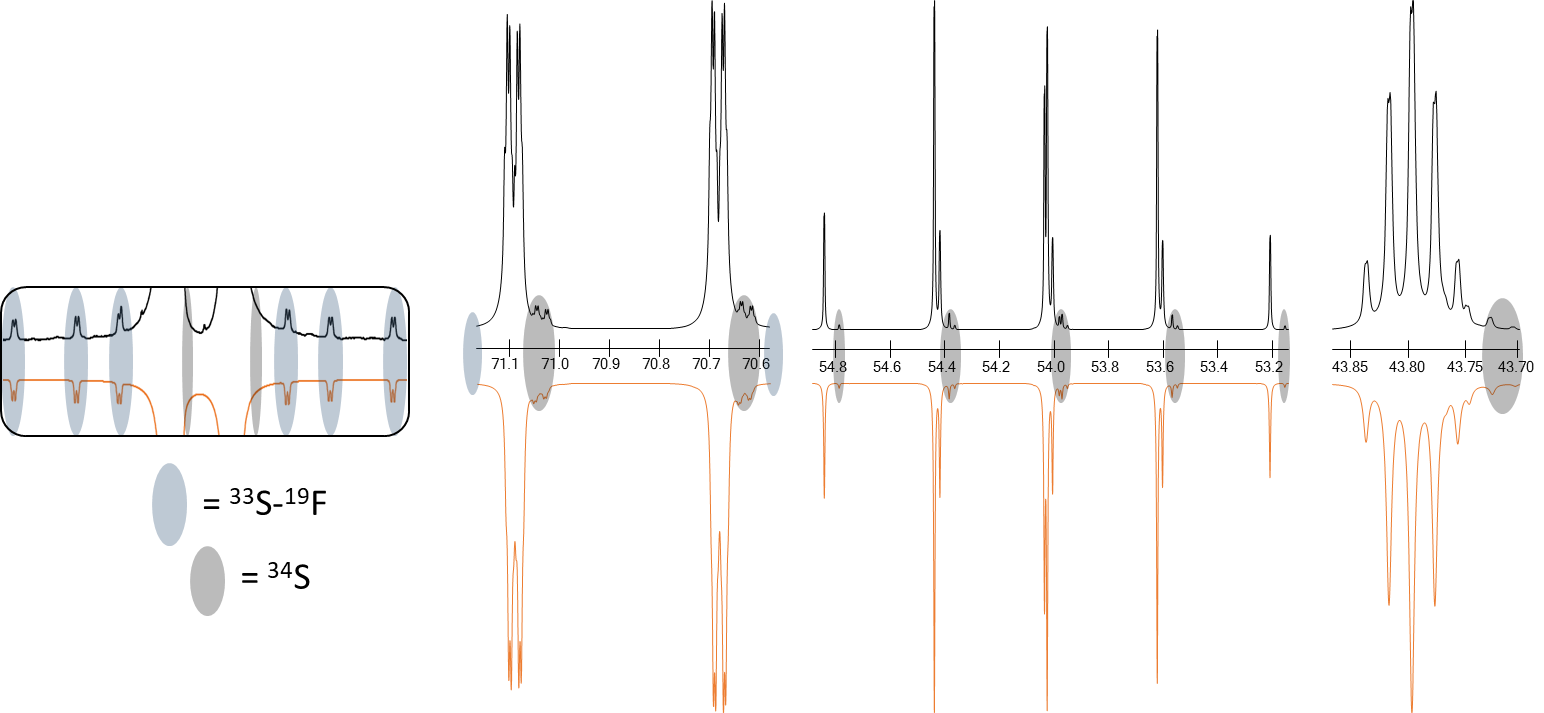** |
| **Figure S11.B** Experimental and simulated ^19^F NMR spectra (377 MHz, neat, r.t.) of FSO_3_SF_5_ (**3**) |

| 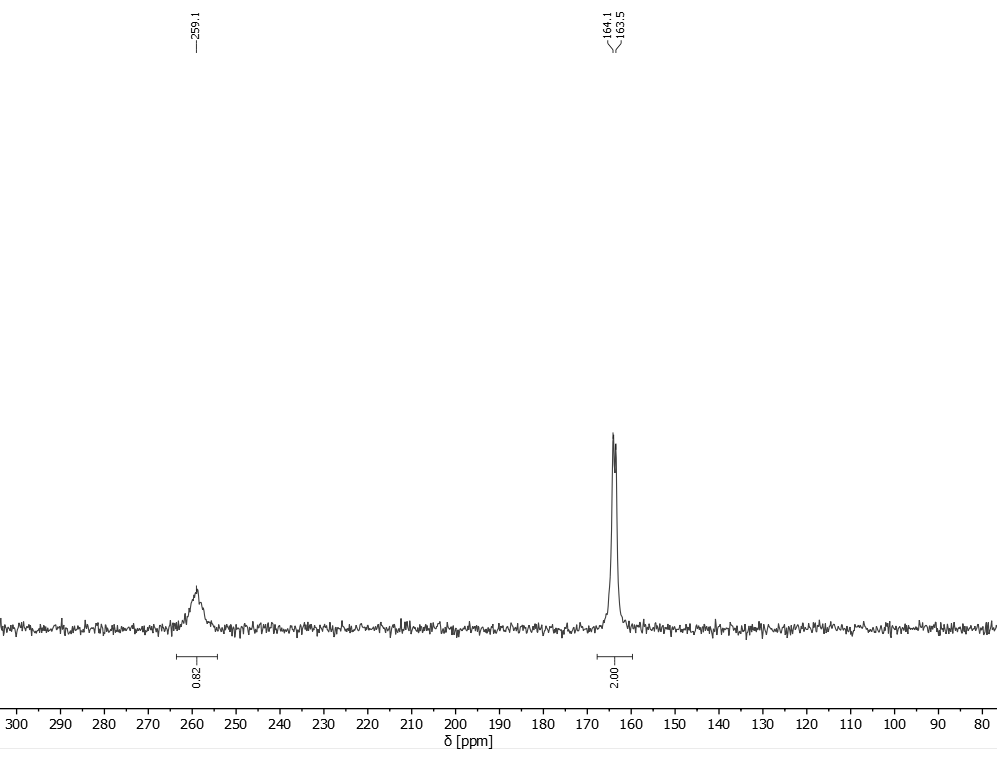 |
| --- |
| **Figure S12.A** Experimental ^17^O NMR spectrum (54 MHz, neat, r.t.) of FSO_3_SF_5_ (**3**) |
| **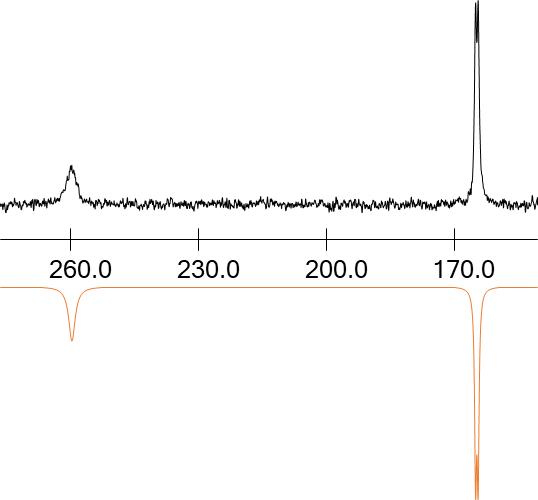** |
| **Figure S12.B** Experimental and simulated ^17^O NMR spectra (54 MHz, neat, r.t.) of FSO_3_SF_5_ (**3**) |

| 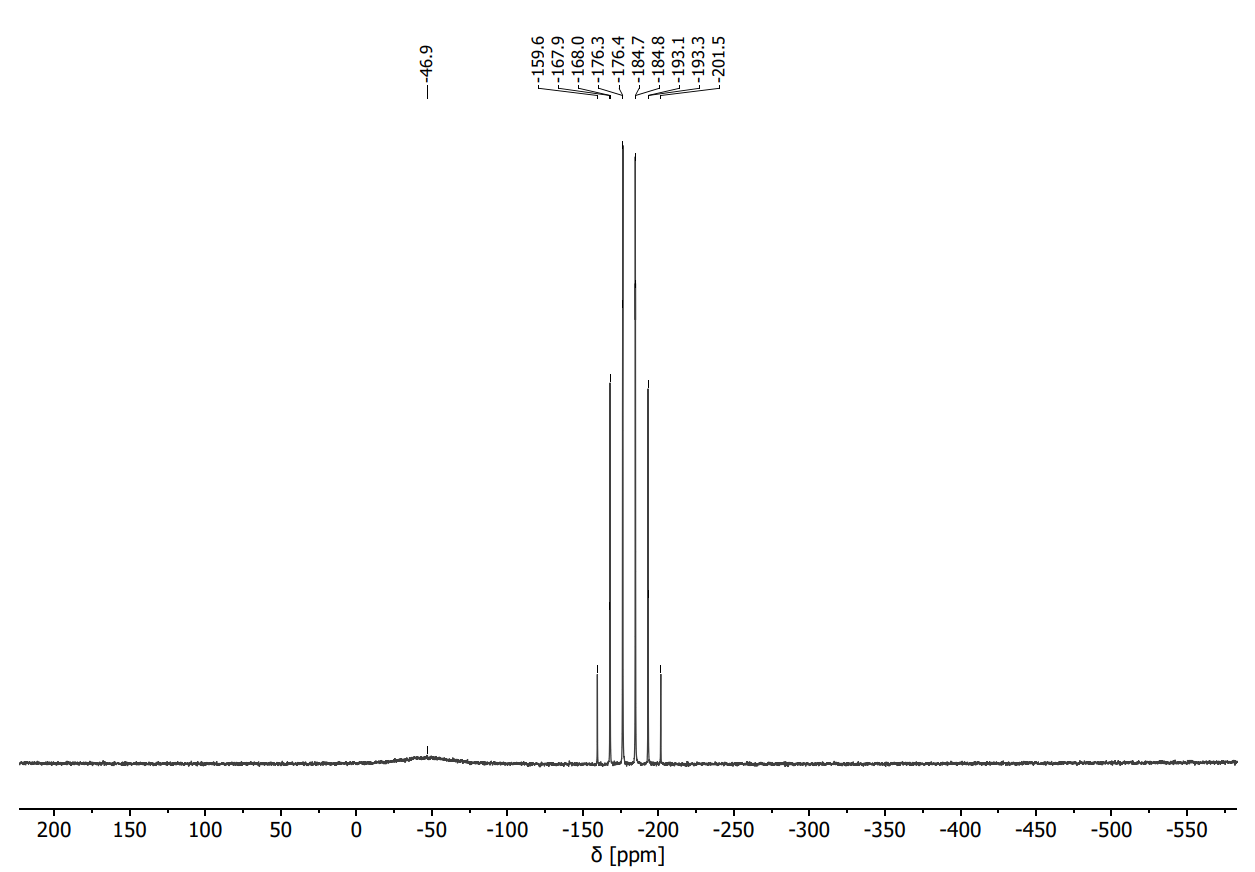 |
| --- |
| **Figure S13.A** Experimental ^33^S NMR spectrum (31 MHz, neat, r.t.) of FSO_3_SF_5_ (**3**) |
| **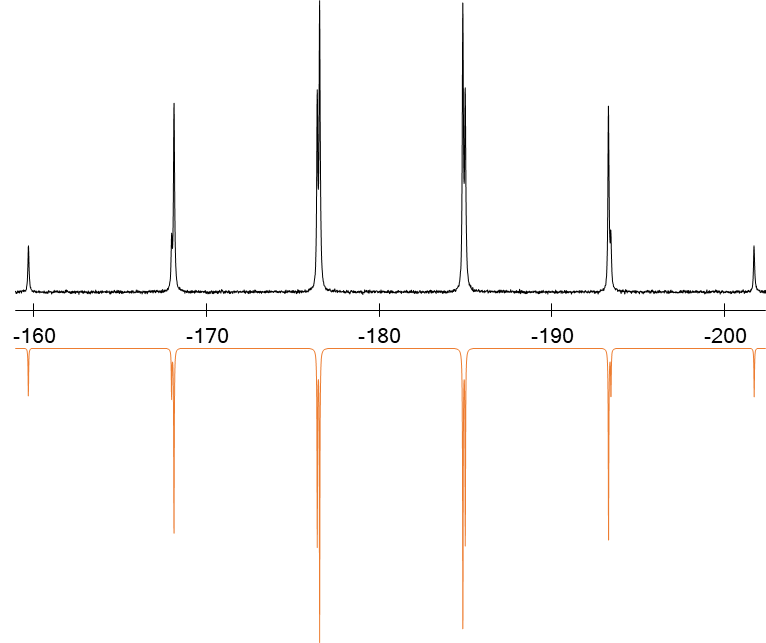** |
| **Figure S13.B** Experimental and simulated ^33^S NMR spectra (31 MHz, neat, r.t.) of FSO_3_SF_5_ (**3**) |
| 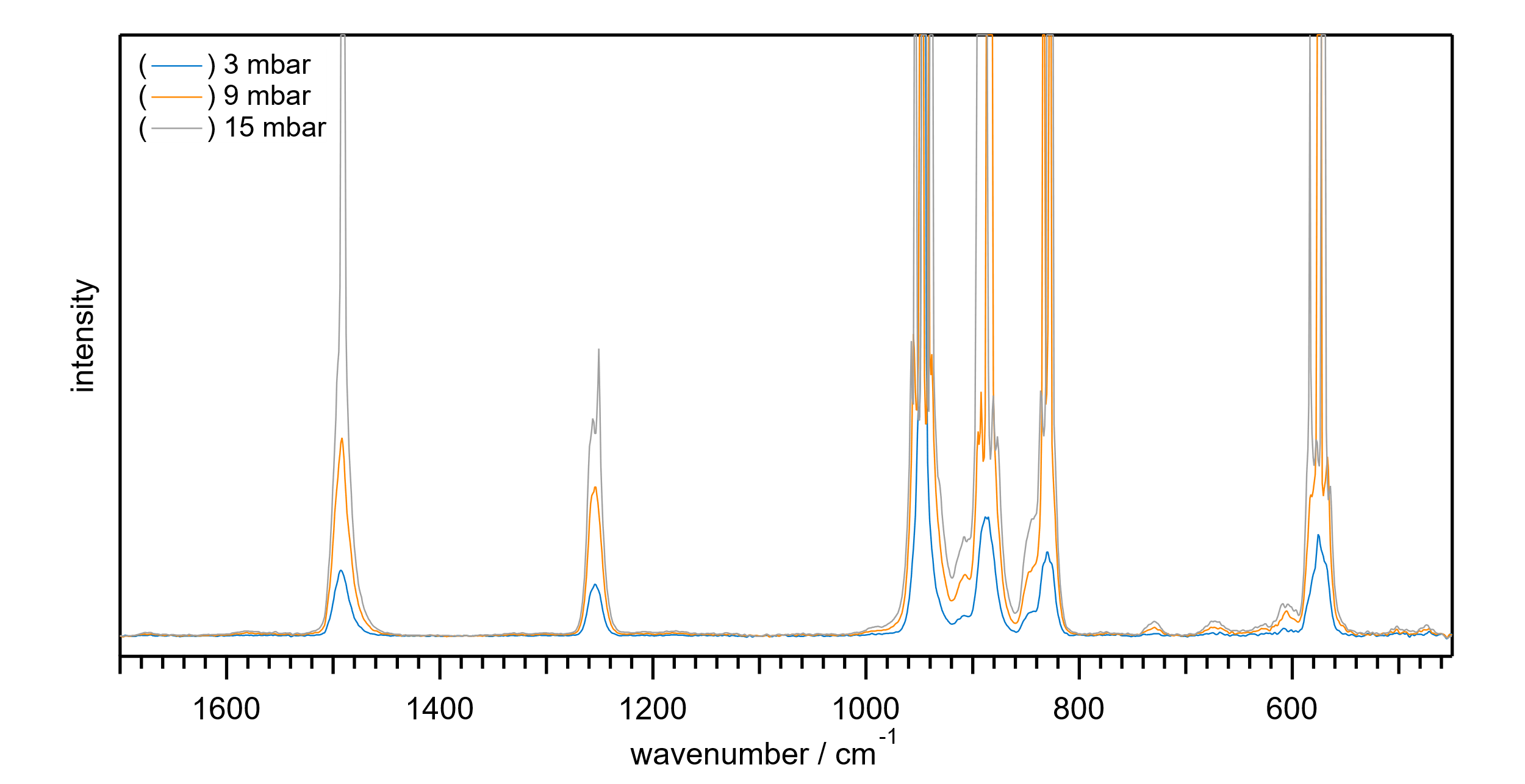 |
| **Figure S14.** Experimental IR spectra of FSO_3_SF_5_ (**3**) |

| 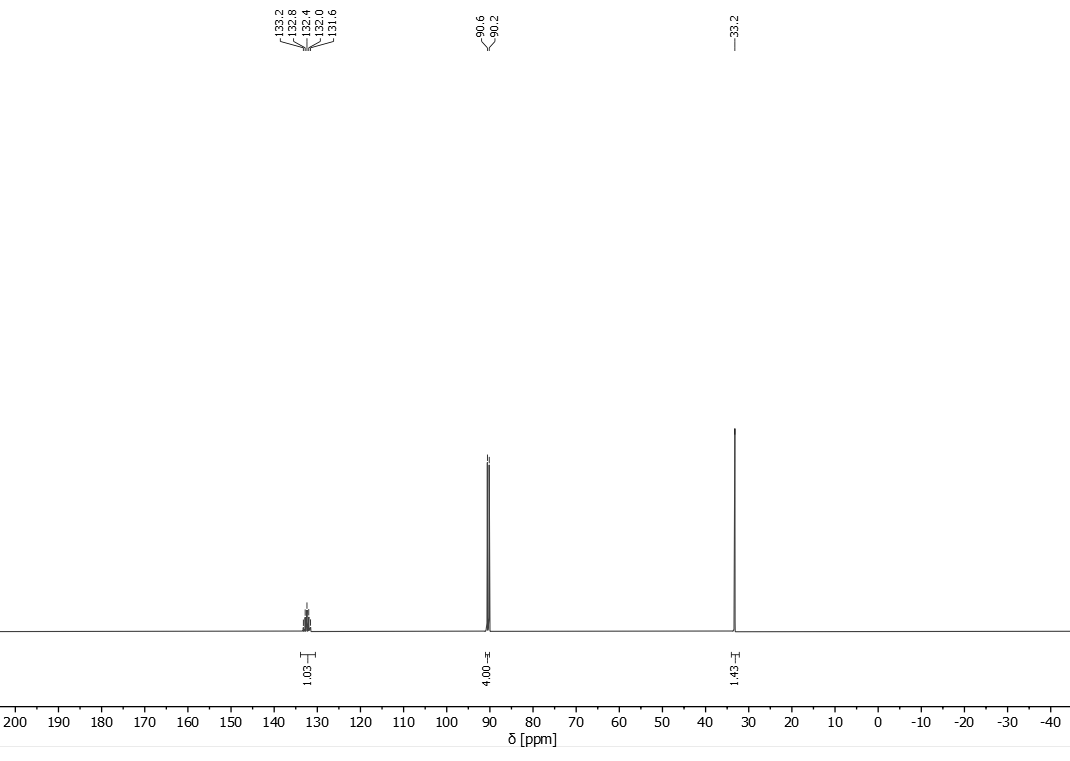 |
| --- |
| **Figure S15.** Experimental ^19^F NMR spectrum (377 MHz, acetone-d6, r.t.) of FSO_3_SF_5_ (**3**) with CsF |

| 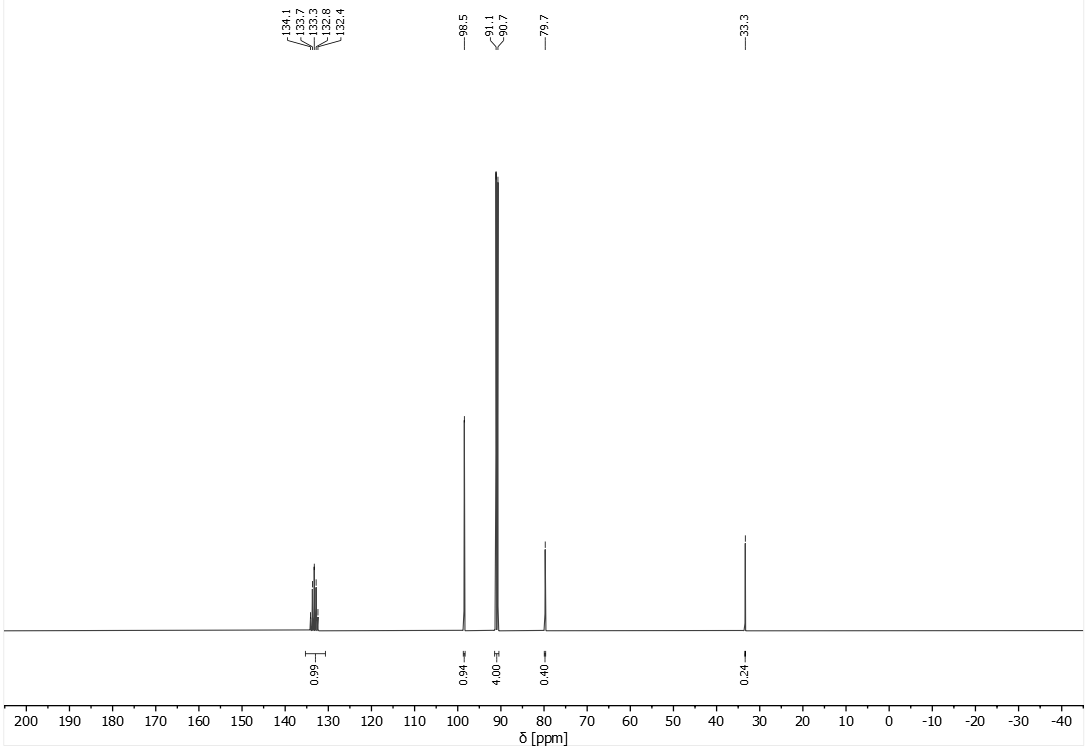 |
| --- |
| **Figure S16** Experimental ^19^F NMR spectrum (377 MHz, CD_2_Cl_2_, r.t.) of FSO_3_SF_5_ with [NEt_3_Me]Cl. SO_2_ClF (98.5 ppm), SOF_4_ (79.7 ppm), SO_2_F_2_ (33.3 ppm). |
| 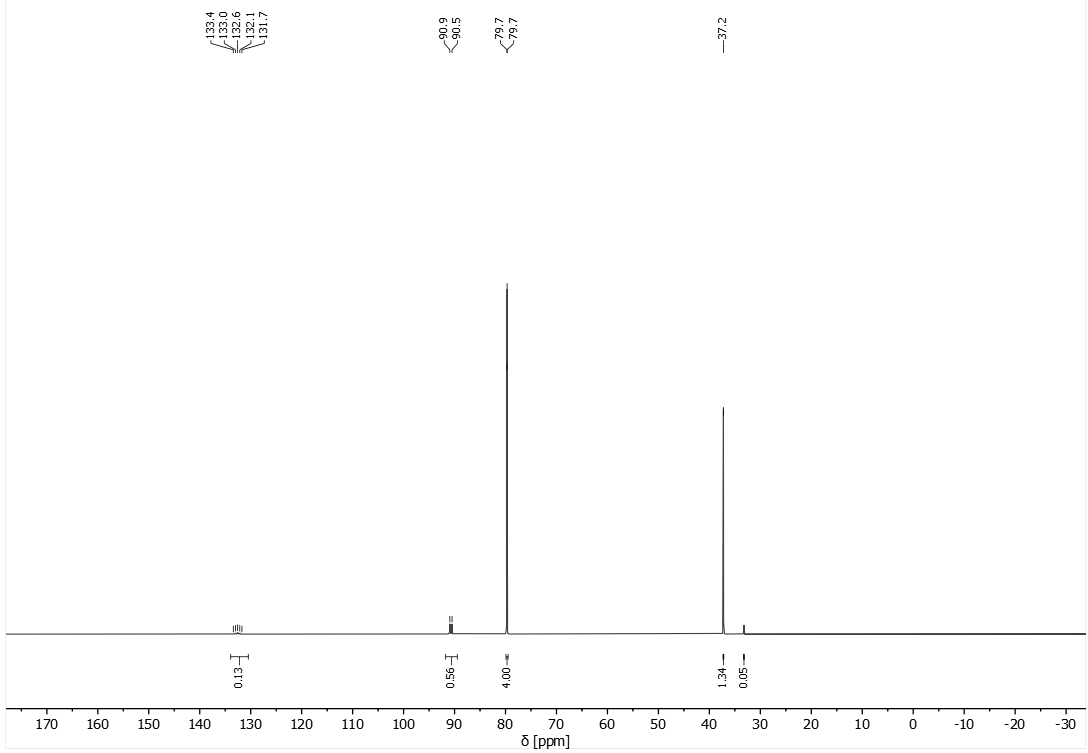 |
| **Figure S17.** Experimental ^19^F NMR spectrum (377 MHz, acetone-d6/DMF r.t.) of FSO_3_SF_5_ with 20% NaF. (SO_2_F_2_ 33.2 ppm) |
| 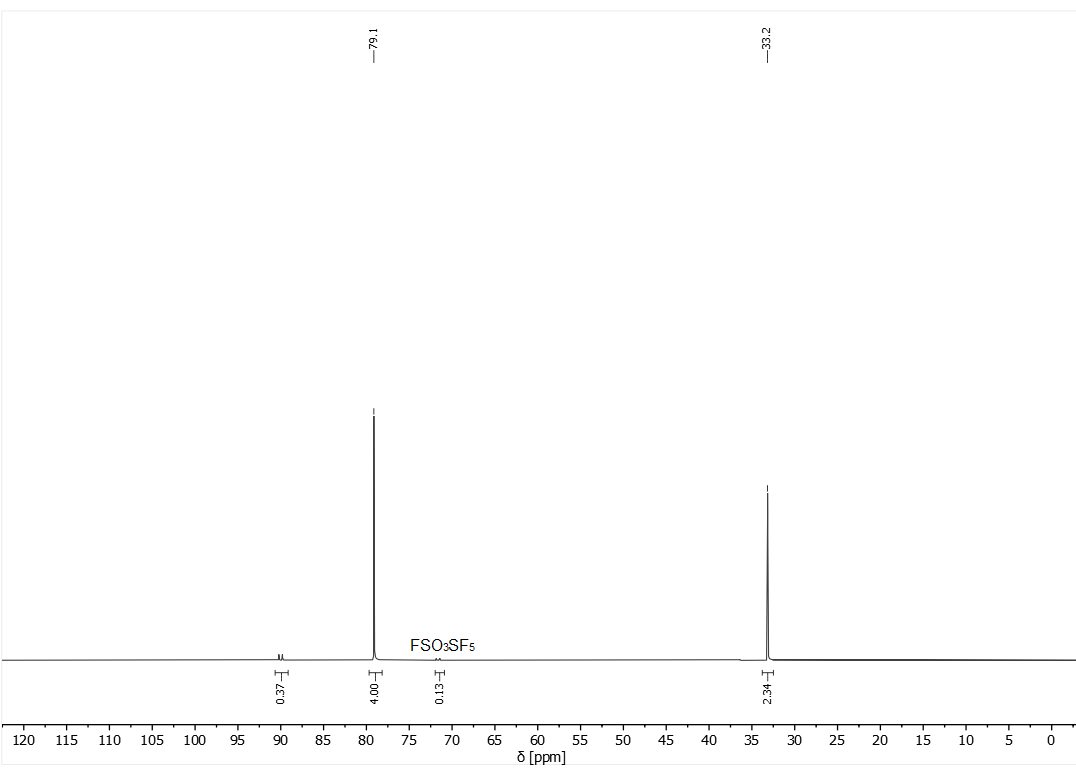 |
| **Figure S18.** Experimental ^19^F NMR spectrum (377 MHz, CD_3_CN, r.t.) of FSO_3_SF_5_ with NaF. OSF_5_ anion (d, 90 ppm) |
| 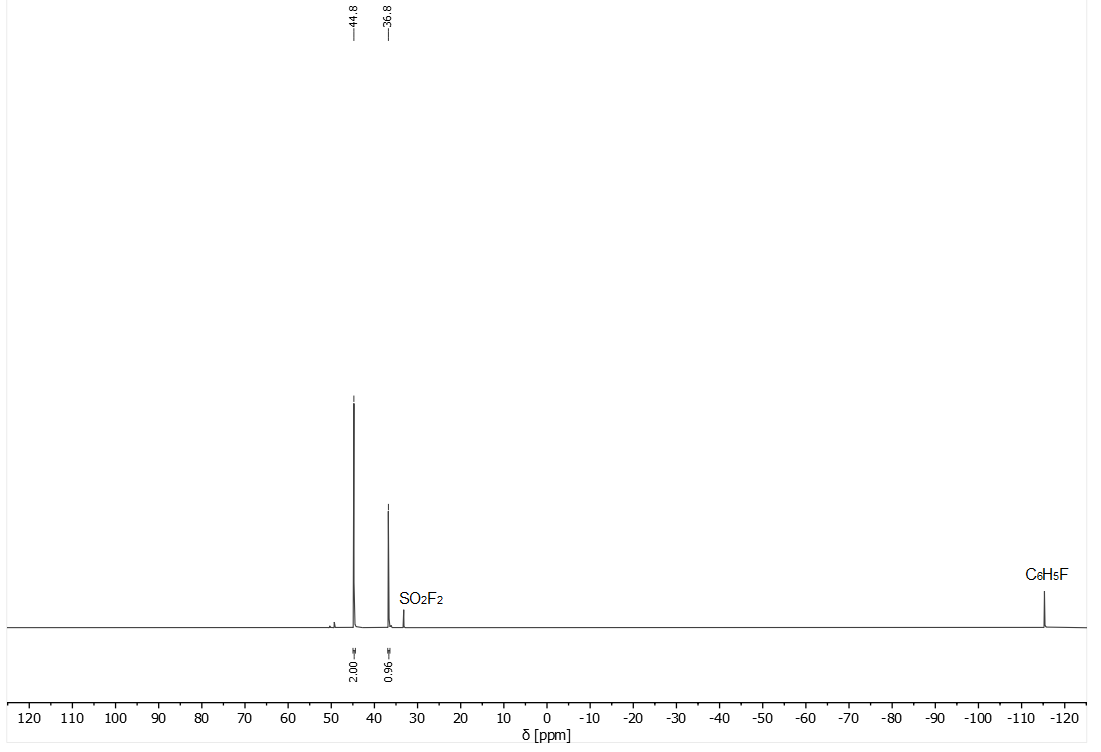 |
| **Figure S19.** Experimental ^19^F NMR crude spectrum (377 MHz, CD_3_CN, r.t.) of **15**. Traces of R-NH-SO_2_F at 50 ppm.   \| 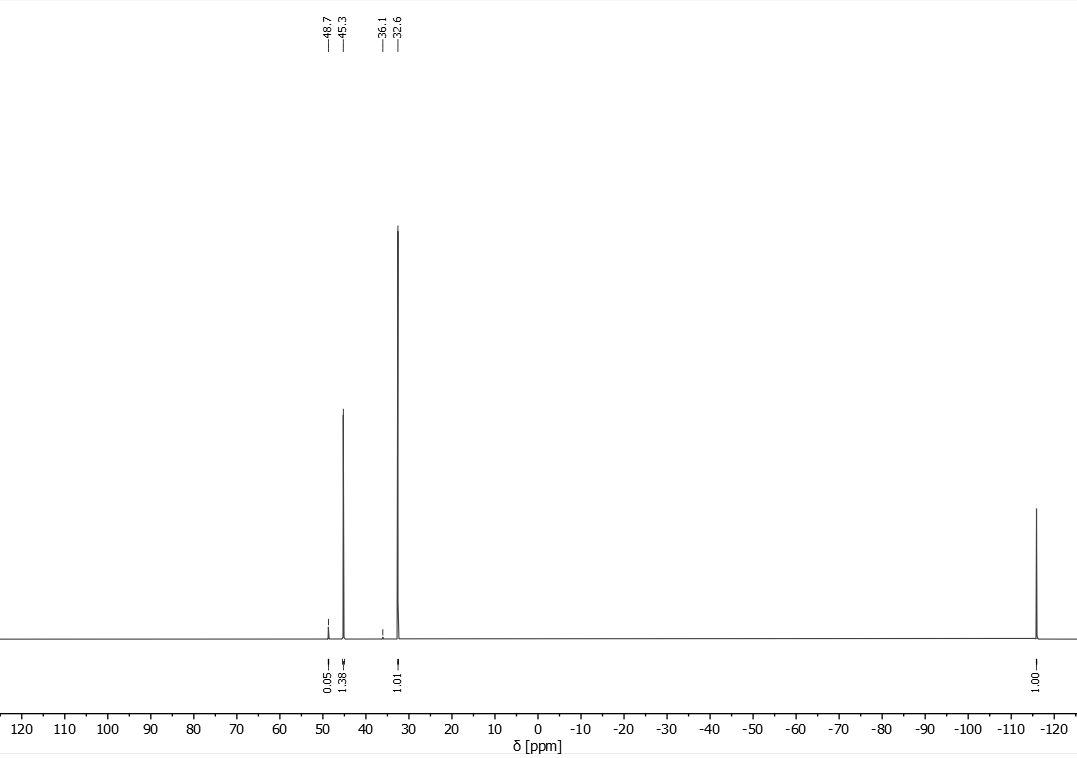 \| \| --- \| \| **Figure S20.** Experimental ^19^F NMR crude spectrum (377 MHz, CH_3_CN, r.t.) of **17** (45.3 ppm). BnNHSO_2_F (48.7 ppm), SO_2_F_2_ (32.6 ppm), traces of possible FSO_3_Na (36.1 ppm). \| \| 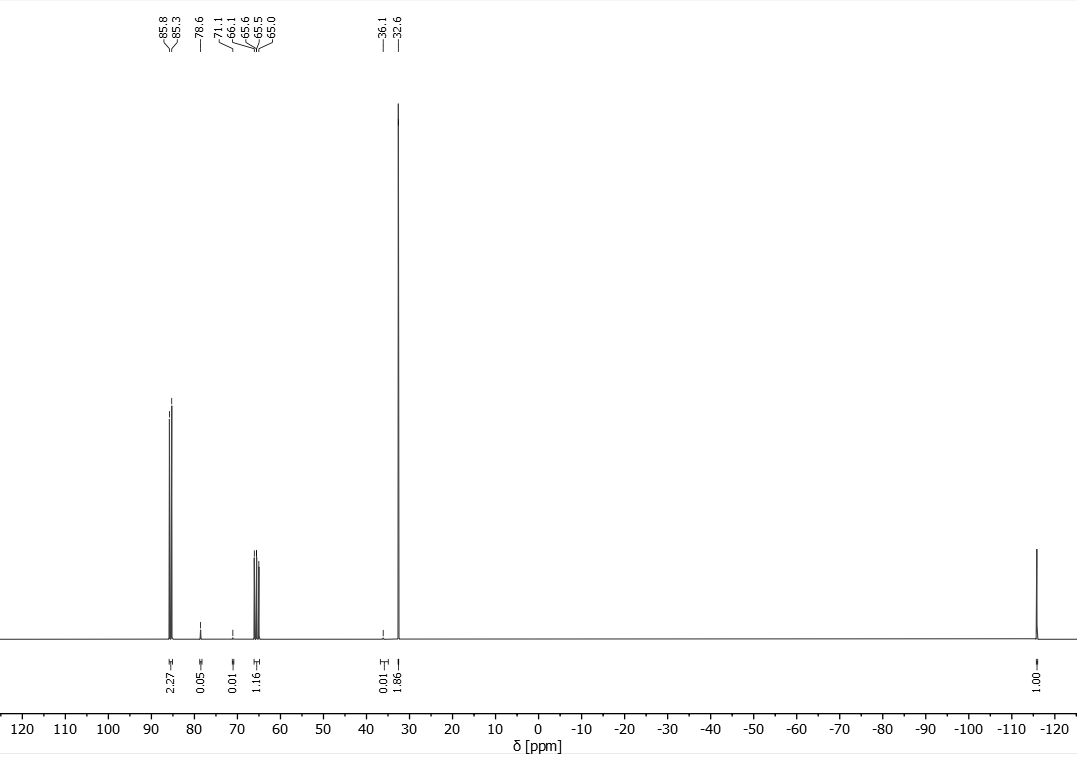 \| \| **Figure S21.A** Experimental ^19^F NMR crude spectrum (377 MHz, CH_3_CN, r.t.) of **19.** SOF_4_ (78.6 ppm), SOF_2_ (71.1 ppm), SO_2_F_2_ (32.6 ppm), traces of possible FSO_3_Na (36.1 ppm). \| \| **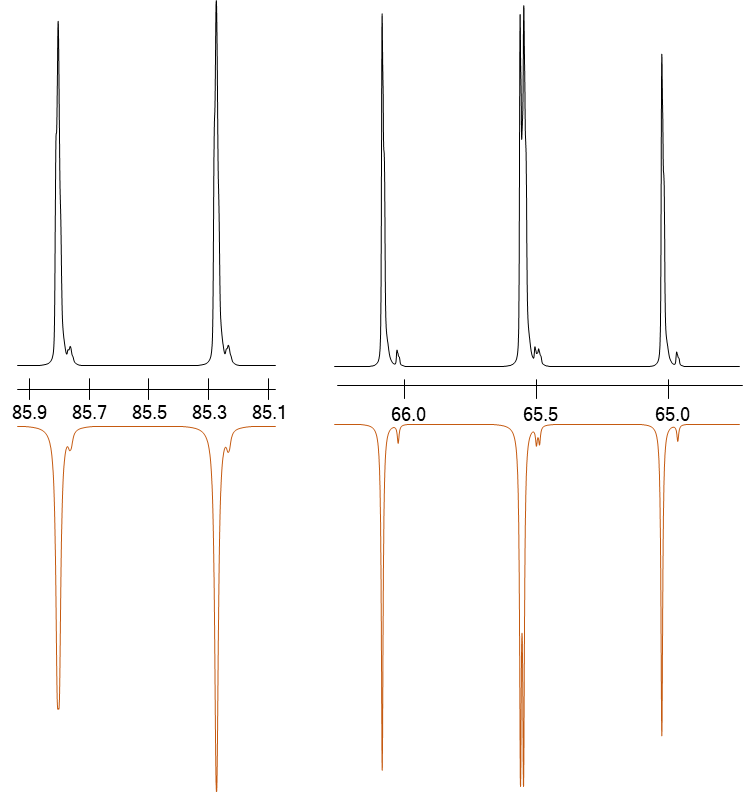** \| \| **Figure S21.B** Experimental and simulated ^19^F NMR (377 MHz, CH_3_CN, r.t.) of **19** from the crude spectrum. \| |

#

# Computational Studies

Structure optimization was performed using Orca V.6.0.1^[16]^ with the B3LYP hybrid functional^[17]^ and the triple-ζ basis set def2-TZVP^[18]^. Minimum structures were confirmed by frequency analysis and were also used for the representation of calculated spectra. NBO analyses were performed on the DFT densities calculated at the DFT minimum structures with the NBO 7.0.^[19]^ Depiction was performed using Chemcraft.^[20]^

**Table S1. Cartesian coordinates of the optimized structures**

| **FOSF_5_** | **FOTeF_5_** | **FSO_3_SF_5_** |
| --- | --- | --- |
| **** | **** | **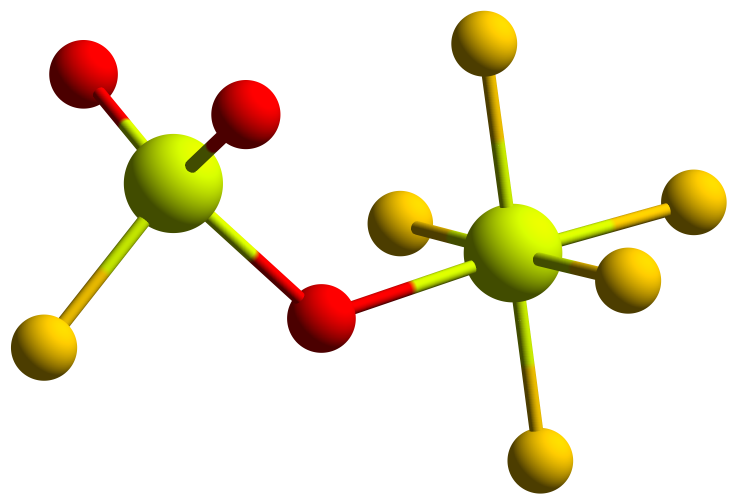** |
| **F 2.13204427494641 -0.45020368730712 -1.23591629405152**  **O 2.02514963005103 0.06252632238540 0.07649391795613**  **S 2.68089134638489 -1.07162112727841 1.18530746779902**  **F 1.38220022052635 -0.84267610482542 2.08419737061930**  **F 1.96693764453241 -2.29333508808109 0.45809689364649**  **F 3.26960089902679 -2.05891039788065 2.28018662714990**  **F 3.98016838192971 -1.28327540296647 0.29255584058649**  **F 3.37868760260240 0.16293548595376 1.91874817629420** | **F 2.06540631426948 -0.37395261214495 -1.38538476638376**  **O 1.94660783293035 0.20793955379582 -0.09648895430576**  **Te 2.68826736176649 -1.07903221848176 1.20475659141568**  **F 1.17636698775364 -0.81057728191528 2.24550862904126**  **F 1.86531431080392 -2.51301168695457 0.36664292846404**  **F 3.36970303801498 -2.21974656504341 2.48453268628970**  **F 4.21000111164067 -1.34250632198038 0.17977022796435**  **F 3.49400304282045 0.35632713272452 2.06033265751449** | **O -5.38863458462637 0.46196512525420 1.16402335151368**  **S -4.95930353760895 -0.06168378128525 -0.07644397323298**  **O -3.75640472086742 -1.12769834266258 0.15237830034255**  **S -2.16933070741850 -0.75943440367612 0.55423270446812**  **F -1.81737384229986 -0.56292412535946 -0.97339864826024**  **F -2.47537501825684 0.79104735797239 0.67762685149631**  **F -0.66170441835329 -0.49083124469582 0.92837570684846**  **F -2.51086711980683 -0.94048289570049 2.08629490770568**  **F -1.87807255660697 -2.30028831041085 0.43198786636883**  **F -5.95363032745693 -1.18711149448074 -0.47996591860434**  **O -4.70813316669801 0.72284211504473 -1.22483114864608** |
| **** | **** | **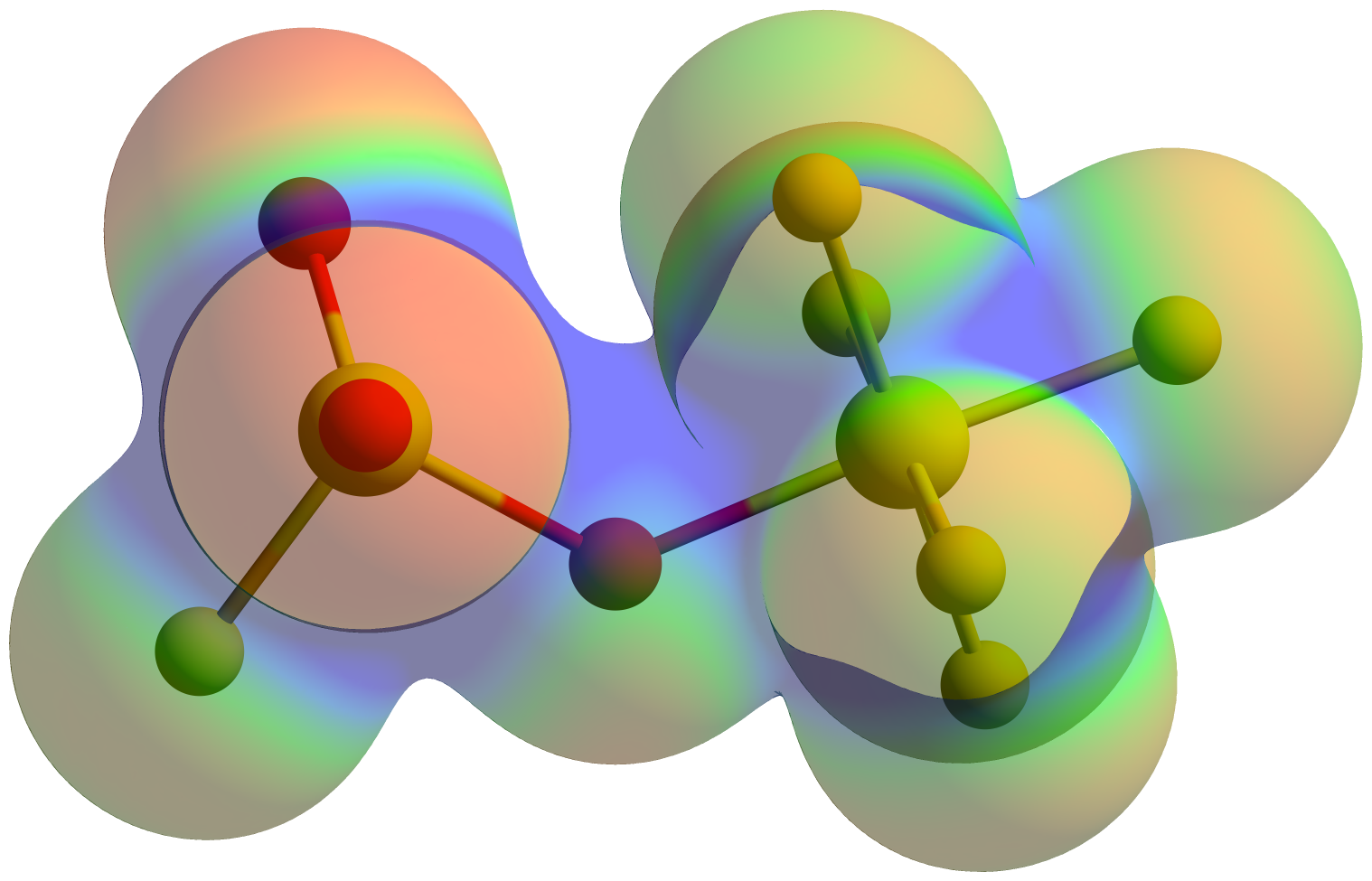** |
| **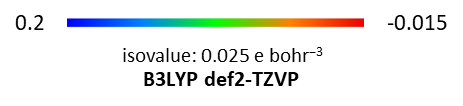** | | |

# References

[1] Mestrelab Research S.L., MestReNova, Mestrelab Research S.L., 2021.

[2] R. J. P. Corriu, J. J. E. Moreau, M. Pataud-Sat, J. Org. Chem. 1990, 55, 2878.

[3] A. Kornath, F. Neumann, Inorg. Chem. 1997, 36, 2708.

[4] J, K, Ruff, C. R, in Inorganic Syntheses, John Wiley & Sons, Ltd, Wiley, 1968, pp. 131–137.

[5] G. Pass, H. L. Roberts, Inorg. Chem. 1963, 2, 1016.

[6] S. M. Williamson, G. H. Cady, Inorg. Chem. 1962, 1, 673.

[7] F. B. Dudley, G. H. Cady, D. F. Eggers, J. Am. Chem. Soc. 1956, 78, 1553.

[8] K. Seppelt, Z. Anorg. Allg. Chem. 1977, 428, 35.

[9] C. Leibold, H. Oberhammer, T. D. Thomas, L. J. Saethre, R. Winter, G. L. Gard, Inorg. Chem. 2004, 43, 3942.

[10] J. S. Sturm, A. Millanvois, C. Bahri, P. Golz, N. Limberg, A. Wiesner, S. Riedel, Chem. - Eur. J. 2024, 30, e202403365.

[11] J.-Y. Shou, F.-L. Qing, Org. Lett. 2025, 27, 2815.

[12] S. Li, P. Wu, J. E. Moses, K. B. Sharpless, Angew. Chem. Int. Ed. 2017, 56, 2903.

[13] S. Li, P. Wu, J. E. Moses, K. B. Sharpless, Angew. Chem. 2017, 129, 2949.

[14] T. Guo, G. Meng, X. Zhan, Q. Yang, T. Ma, L. Xu, K. B. Sharpless, J. Dong, Angew. Chem. Int. Ed. 2018, 57, 2605.

[15] D. S. Ross, D. W. A. Sharp, J. Chem. Soc., Dalton Trans. 1972, 34.

[16] F. Neese, F. Wennmohs, U. Becker, C. Riplinger, The Journal of chemical physics 2020, 152, 224108.

[17] a) C. Lee, W. Yang, R. G. Parr, Physical review. B, Condensed matter 1988, 37, 785; b) A. D. Becke, Physical review. A, General physics 1988, 38, 3098.

[18] a) F. Weigend, Physical chemistry chemical physics : PCCP 2006, 8, 1057; b) F. Weigend, R. Ahlrichs, Physical chemistry chemical physics : PCCP 2005, 7, 3297.

[19] E. D. Glendening, C. R. Landis, F. Weinhold, Journal of computational chemistry 2019, 40, 2234.

[20] Chemcraft - graphical software for visualization of quantum chemistry computations. Version 1.8, build 682. https://www.chemcraftprog.com.

# Author Contributions

S.R. and A.M. conceived the project and A.M. designed the research. A.M.; C.B.; T.D. conducted the experiments and analyses, A.M. performed the computational studies; S.S. and A.M. performed the NMR analysis. The first draft and first review were respectively done by A.M. and C.B. then all authors took part in the reviewing of the manuscript.
